# Antimicrobial and Antiviral (SARS-CoV-2) Potential of Cannabinoids and *Cannabis sativa*: A Comprehensive Review

**DOI:** 10.3390/molecules26237216

**Published:** 2021-11-28

**Authors:** Md Sultan Mahmud, Mohammad Sorowar Hossain, A. T. M. Faiz Ahmed, Md Zahidul Islam, Md Emdad Sarker, Md Reajul Islam

**Affiliations:** 1 Faculty of Textile Engineering, Bangladesh University of Textiles, Dhaka 1208, Bangladesh; sultan.mahmud@ye.butex.edu.bd (M.S.M.); a.t.m.faiz@fe.butex.edu.bd (A.T.M.F.A.); zahid@ye.butex.edu.bd (M.Z.I.); 2 Biomedical Research Foundation, Dhaka 1230, Bangladesh; sorowar.hossain@brfbd.org; 3 School of Environment and Life Sciences, Independent University, Dhaka 1229, Bangladesh

**Keywords:** antibiotic resistance, antimicrobial, cannabinoid, cannabis, COVID-19, food-borne, plant pathogen

## Abstract

Antimicrobial resistance has emerged as a global health crisis and, therefore, new drug discovery is a paramount need. *Cannabis sativa* contains hundreds of chemical constituents produced by secondary metabolism, exerting outstanding antimicrobial, antiviral, and therapeutic properties. This paper comprehensively reviews the antimicrobial and antiviral (particularly against SARS-CoV-2) properties of *C. sativa* with the potential for new antibiotic drug and/or natural antimicrobial agents for industrial or agricultural use, and their therapeutic potential against the newly emerged coronavirus disease (COVID-19). Cannabis compounds have good potential as drug candidates for new antibiotics, even for some of the WHO’s current priority list of resistant pathogens. Recent studies revealed that cannabinoids seem to have stable conformations with the binding pocket of the M^pro^ enzyme of SARS-CoV-2, which has a pivotal role in viral replication and transcription. They are found to be suppressive of viral entry and viral activation by downregulating the ACE2 receptor and TMPRSS2 enzymes in the host cellular system. The therapeutic potential of cannabinoids as anti-inflammatory compounds is hypothesized for the treatment of COVID-19. However, more systemic investigations are warranted to establish the best efficacy and their toxic effects, followed by preclinical trials on a large number of participants.

## 1. Introduction

**Figure 1:**
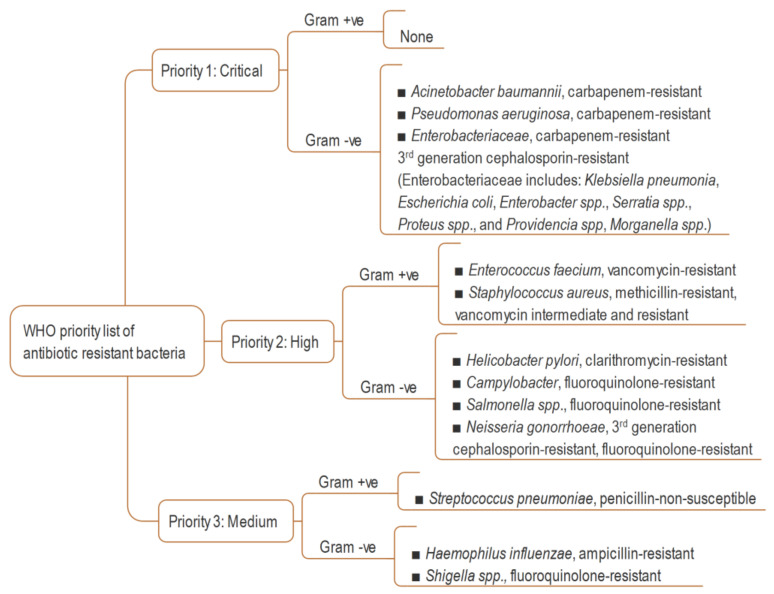
WHO global priority list of resistant bacteria [15].

The term ‘antimicrobial agent’ refers to specific synthetic or natural substances such as drugs, chemicals, or extracts that have the ability to either kill or inhibit the growth of microbes, including bacteria, fungi and algae [1]. Antibiotics have played a tremendous role in attenuating mortality and morbidity of humans since the antibiotic era started at the early of the last century [2,3]. The introduction of antibiotics into therapeutics has extended the average human life expectancy by around 23 years in just 100 years [4]. However, because of widespread misuse of antibiotics, bacteria have developed mechanisms to escape from antimicrobial agents. Although antibiotic resistance is a natural phenomenon [5] (it was observed before the extensive use of penicillin [6]), its pace has been accelerated due to overuse, inappropriate prescribing and extensive agricultural use [7]. Today, antimicrobial resistance is one of the greatest challenges for global health, and the World Health Organization (WHO) has declared it one of the top threats for humanity [8]. In the United States, more than 2.8 million people are infected by antibiotic-resistant bacteria, with over 35,000 deaths every year. An estimated USD $4.6 billion is spent to fight only six multidrug-resistant pathogens [9]. Globally, drug resistant infections cause half a million deaths each year, and the toll is suspected to exceed 10 million by 2050 [10]. Many first-line antibiotics are predicted to be ineffective by 2025 and, consequently, the ‘post antibiotic era’ will start soon, or may already has started [9,11]. Though the discovery of new antibiotics is critical, concerning the pace of antibiotic resistance, unfortunately, a huge innovation gap has been created in antibiotic drug discovery after the end of its ‘golden era’ between 1950 and 1970 [12]. It is almost 50 years since the last new antibiotic was discovered, and research funding to find new antibiotics has been drastically reduced in both the pharmaceutical and academia domain, which considering such investment nonprofitable during an economic crisis [13,14]. In 2017, the WHO published a global priority pathogen list comprising 12 species of bacteria categorized by critical, high, and medium antibiotic resistance, with the aim of ensuring quick R&D responses, guiding strategic directions and achieving new antibiotics for urgent public health needs (Figure 1) [15]. The United States Centers for Disease Control and Prevention’s (CDC) 2019 AR Threats Report listed 18 germs, including bacteria and fungi, on three levels of human health concern: urgent, serious, and concerning, as a measure of estimation of antibiotic resistance burden in the USA [9]. Today, the world is witnessing how an emerging infectious disease such as the COVID-19 pandemic, caused by Severe Acute Respiratory Syndrome Coronavirus 2 (SARS-CoV-2), can result from a lack of appropriate medicines, in addition to many other causes. The pandemic led to more than 4.8 million documented deaths globally in the 23 months up to 6 October 2021 [16].

In the history of the treatment of infectious diseases, cannabis has been used for thousands of years without knowledge of the scientific background of its effects [17,18]. A substantial amount of research has documented that *C. sativa* possesses hundreds of secondary metabolites including cannabinoids, terpenes and phenolic compounds [19] which have pharmacological properties in anticonvulsant therapy, appetite stimulation, neurodegenerative diseases, pain treatment, skin pathologies and infectious diseases [20]. Cannabinoids and terpenes, or essential oils (EO) enriched with these, are well known to confer anti-inflammatory effects in mammals during infectious diseases [21,22,23]. So far, 545–550 known compounds, of which about 177 phytocannabinoids, about 200 terpenes and nearly same number of phenolics, have been identified from *C. sativa* [20,24,25,26]. Bonini et al. reviewed the pharmacological potential of cannabinoids, stating that preclinical and clinical studies of cannabinoid compounds are beneficial for treatment of pain, colitis, spasticity, nausea and vomiting, anorexia, sleep disorders, anxiety, epilepsy, and Alzheimer’s disease [24]. Since cannabinoids can modulate the immune response through binding CB1 and CB2 receptors (a G-protein-coupled receptor densely located in the immune tissue, nervous tissue and brain), their role in infectious diseases has been discussed critically in many scientific publications [27,28,29,30,31,32]. However, the antimicrobial activity of cannabinoids, extracts and EOs from *C. sativa* is not unexpected, as many secondary metabolites of plants exhibit bioactivity against numerous pathogenic bacteria and fungi [33,34,35]. There is also fragmentary evidence in the literatures that cannabis compounds have efficacy against some viruses [25,32]. This paper represents a comprehensive review of the antibacterial, antifungal, antiviral, and therapeutic potential for cannabinoids, cannabis extracts and EOs against COVID-19, based on research in old and contemporary articles. The literature reviewed demonstrate the broad spectrum of hemp’s antibacterial activity, with the goal of showing the plant’s utility for multipurpose antibacterial applications.

## 2. Antibacterial Activity of Cannabinoids and *C. sativa*

### 2.1. Historical Overview

The antibacterial efficacy of *C. sativa* was scientifically revealed in a dissertation by Krejci in 1950 [36] and preliminary results were published later stating that extracts were effective against only Gram-positive bacteria (GPB) [37,38]. Independently, the microbial inhibitory property of seeds of hemp was observed by Ferenczy in 1956. The diffused compounds from whole seeds produced an inhibitory zone against GPB in culture medium [39]. Later, resinous organs of the plant, such as the seeds and leaves, exhibited a considerable amount of antibacterial activity against GPB in an acidic culture medium, but were found ineffective against gram negative bacteria (GNB), yeasts and molds [40]. It was observed that the antibacterial activity depended on the intensity of the hashish reaction, which indicated the activity might come from psychoactive Δ^9^-tetrahydrocannabinol (THC), though other cannabinoids from *C. sativa* had not been identified at that time [40]. The following sections include some subsections of the WHO priority list, as well as some non-listed pathogenic bacteria.

### 2.2. Antibacterial Activities of Cannabinoids against Pathogens in the WHO’s Priority List

Cannabinoids and *C. sativa* extracts have substantial activity against several resistant bacteria in the WHO’s current priority list (Table 1). All major cannabinoids, including cannabidiol (CBD), THC, cannabigerol (CBG), cannabichromene (CBC), cannabinol (CBN), their derivatives like cannabidiolic acid (CBDA), cannabichromenic acid (CBCA), and even extracts and EOs, inhibit MRSA including the epidemic-causing EMRSA 15 and EMRSA 16. Methicillin-resistant Staphylococcus aureus (MRSA) are resistant to all known beta-lactam antibiotics [41], and even to linezolid, daptomycin and vancomycin [42]. Extensive work has been published recently by Farha et al., enlightening the antibiotic potency of major cannabinoids against MRSA regarding their efficacy to inhibit biofilms and persister cells [43]. Biofilms represent a subpopulation of bacteria that secure themselves against adverse situations, and persister cells, which are dormant and non-dividing, are common sources of antibiotic tolerance to MRSA [44,45]. When a biofilm forms, bacterial cells acquire 10–1000 times more resistance to antibiotics [46]. Biofilms and persisters of MRSA are considered important virulence factors, especially when formed on necrotic tissues and medical devices [43]. All five major cannabinoids can obstruct the formation of biofilms, destroy preformed biofilms and eradicate stationary phase cells of MRSA. MRSA persisters, which are highly resistant to gentamicin, ciprofloxacin, and vancomycin [47] can be killed by cannabinoids, and notably by CBG, at a concentration of 5 µg/mL [43], whereas oxacillin and vancomycin are ineffective [48]. The MIC_90_ of CBG against MRSA strains is favorable compared to conventional antibiotics [43]. The efficacy of CBG against biofilms and persisters of MRSA was found to be MIC 2 µg/mL in vivo, in a murine systemic infection model. CBG was found to be hemolytic at only 32 µg/mL, many-fold higher than MIC [43].

The rapid bactericidal activity of CBD was observed (<3 h) at 2 µg/mL [49], and the effect resembled that of the natural nonionic detergents, saponins [50]. CBD and CBDA showed no toxicity to human keratinocyte cells at up to seven and four-fold higher concentration of their respective MIC against MRSA (Table 1) [50]. CBD could potentiate bacitracin activity, reducing its MIC 64-fold against resistant bacteria, including MRSA [51]. The combination affected morphological changes of the pathogen, impaired cell division and induced membrane irregularities. No synergistic or antagonist effect was seen on MRSA resulting from CBD with conventional antibiotics including vancomycin, methicillin, clindamycin, tobramycin, teicoplanin, ofloxacin and meropenem [50]. Because of the hydrophobic nature of CBD, it cannot attack enough of the bacterial membrane to enhance the uptake of antibiotic drugs and does not interfere the mechanism of action of last-resort antibiotics.

In an in vivo study, CBCA showed more potent and faster bactericidal activity than vancomycin with lower a toxicity level to the mammalian cell lines A549 and HepG2. CBCA and cannabidivarin methyl ester (CBDVM) rendered minimum toxicity concentration (MTC), greater than 100 µM on both cell lines, which is far higher than their respective MIC against MRSA (Table 1). Additionally, compared to vancomycin, the compound exhibited more biocidal activity with higher a bacterial load. Rapid bactericidal activity of CBCA could reduce treatment time and provide less opportunity for emergence of bacterial resistance. A time-kill assay showed considerable reduction of CBCA activity after 8 h of exposition to MRSA. The activity of CBCA was observed against both the exponential and stationary phases of MRSA and was independent of their cellular metabolism [52]. The killing activity of many antibiotics is attributed to their effect on dividing bacteria cells, which is crucially interrupted by the stationary phase of MRSA, resulting in higher morbidity in nosocomial infections [53]. Synergistic effects of phytocannabinoids and terpenoids are reported in the treatment of infections related to MRSA and fungi [54]. The penetration of bacteria cell membranes differs among cannabinoids, which results in the non-identical effects of these compounds [50].

In contrast to pure active compounds, *C. sativa* extracts and EOs sometimes have even greater activity against resistant pathogens as a result of probable synergism. Drug-resistant clinical isolates, including MRSA, vancomycin-resistant Staphylococcus aureus (VRSA) and vancomycin-intermediate Staphylococcus aureus (VISA) demonstrated susceptibility to alcoholic *C. sativa* extracts [55,56]. A profound inhibitory efficacy was achieved when an ethanolic extract of *C. sativa* leaves was combined with a Thuja orientalis leaf extract in a 1:1 ratio. The synergism was obtained due to the antibacterial effect of the phenolic compounds quercetin, gallic acid and catechin present in the leaf extract [55].

Gram-negative organisms generally exhibit more resistance to antibiotics due to their distinctive structure. They are dominant killers in intensive care units showing resistance to wide-spectrum antibiotics including third-generation cephalosporins and carbapenems [57]. They differ in structure from GPB since they have an outer membrane containing lipopolysaccharide (LPS)/endotoxin, which provides the pathogen intrinsic resistance against antibacterial agents [58]. This acts as an important barrier and provides protection by resisting the penetration of toxic antibiotics and innate host immune molecules [59].

However, GNB, whose outer membrane is permeable, are susceptible to cannabinoids [43]. All the five major cannabinoids showed synergism against clinically isolated multidrug-resistant GNB, including Klebsiella pneumoniae, Acinetobacter baumannii, Pseudomonas aeruginosa and Escherichia coli when used with polymyxin B at sublethal concentration [43,49]. The activity against *K. pneumoniae* was increased for EO exhibiting full synergism with addition of ciprofloxacin [60]. Naringenin with EO was found to be bactericidal against drug resistant Helicobacter pylori [61]. Aqueous and solvent extracts of leaf, stem and roots also displayed substantial activity against *K. pneumoniae*, *A. baumannii* and Haemophilus influenzae [62].

CBD has strong inhibitory efficacy on release of membrane vesicles (MV) from *E. coli* VCS257 and can boost bactericidal power of vancomycin against *E. coli*, to which it shows resistance [63]. MVs are nanosized spheres composed of lipid membranes derived from the outer membrane of bacteria that can cause an extra layer of protection against antibiotics [64,65]. EO exhibits synergistic effect against *E. coli*, and *P. aeruginosa* in combination with ciprofloxacin [60]. *P. aeruginosa* is resistant to antibiotics including beta-lactams, aminoglycosides and quinolones [66]. The efficacy of solvent extracts of *C. sativa* against *P. aeruginosa* in terms of inhibitory zone is comparable with gentamicin [67], ampicillin [68] and ciprofloxacin [60]. Notably, the level of sensitivity of the extracts in qualitative tests is not equipollent since their polarity and solubility change their diffusivity through media [69,70]. However, in many other investigations, the activity of *C. sativa* was shown against *P. aeruginosa* [62,71,72,73,74,75], *E. coli* [62,67,68,72,76,77,78,79,80,81,82], Salmonella species [76,80,83,84], Shigella species [76,82], *K. pneumoniae* [82], *Acinetobacter calcoaceticus* [79], *Morganella morganii* [62] and *Serratia marcescens* [84].

The ability of cannabinoids to modulate physiological and pathophysiological activities can hinder bacterial conjugation by targeting plasmid DNA [85]. Conjugation is one of the major processes of acquiring antibiotic resistance and involves replication and transfer of an extra piece of bacterial DNA plasmid into a recipient bacterium [86]. Plasmids contain genes to express resistance to antibiotics. Δ^9^-THC, CBN and CBD impaired plasmid transfer activity near to zero for pKM 101 and TP 114 [85]. Tetrahydrocannabinolic acid (THCA) reduced plasmid curing activity by 30% in *E. coli* K12 F’lac strain [87]. Plasmid curing is a process by which the plasmid is eliminated, and the bacteria become susceptible. THCA and some cannabispiro compounds were inhibited transformation of plasmid DNA (pBR322), elimination (F’lac) and transfer (R144) of plasmid from *E. coli* to E. coli, and even killing plasmid carrying bacteria despite possessing a higher MIC value [88].

Apart from phytocannabinoids, some endocannabinoids (EC) and endocannabinoid-like (EC-like) natural endogenous compounds have good potency against MRSA biofilms. Anamide and arachidonoyl serine, an EC and EC-like natural endogenous compound respectively, did not kill the bacteria in vitro, but inhibited biofilm formation and preformed biofilms of MRSA, altered biofilm-associated virulence factors, and could modify MRSA cell surface characteristics [89]. The compounds also exhibited synergy with different antibiotics including ampicillin, methicillin and gentamicin under both planktonic growth conditions and biofilm formation [90]. Besides, their combination with methicillin impaired slime formation of MRSA [90]. The slime layer is not easily be washed off and can be expressed as a virulence factor [91,92].

### 2.3. Antibacterial Activities of Cannabinoids against Pathogenic Bacteria Not on the WHO Priority List

*C. sativa* has broad-spectrum antibacterial efficacy against a number of pathogenic bacteria (Table 2) that are not listed in WHO’s current priority list.

CBD has bacitracin activity, reducing its MIC 64-fold against Listeria monocytogenes and Enterococcus faecalis [51]. It can increase the effectiveness of kanamycin against Staphylococcus aureus without affecting MV release [63]. The EO exhibited bactericidal activity against clinically isolated methicillin-resistant Staphylococcus pseudintermedius (MRSP) from dogs suffering from pyoderma [102]. A combination of ciprofloxacin with EO significantly decreased MIC against Bacillus subtilis, *S. aureus* and *Micrococcus luteus* due to partial and full synergism [60]. The inhibition pattern of seed extract against *S. aureus* biofilms is similar to that of vancomycin, and the efficacy was found to be dose-dependent [103]. The bactericidal activity of solvent extracts against penicillin resistant *S. aureus* was recorded by Kabelik [18,104]. Acidic fractions are responsible for the antimicrobial properties of crude extract of leaves [105]. Leaf extract out-performs chloramphenicol in terms of inhibition zone against the strep-throat-causing Lancefield Group A *Streptococcus* sp., and its activity is comparable with penicillin and amoxicillin [10], which are commercially used as beta-lactam antibiotics for strep-throat treatment.

Moreover, a considerable number of diffusion tests showed medium to higher activity against *S. aureus* [67,68,71,74,76,77,79,82,84,105,106], *B. subtilis* [67,79,80,82,84,105], *Bacillus cereus* [77,80,84], *Bacillus pumilus* [105], *E. faecalis* [77,83,84,107], *Micrococcus flavus* [105], *M. luteus* [79,84], *Brevibacterium linens*, *Brochothrix thermosphacta* [79] and Methicillin-resistant coagulase-negative Staphylococci (MRCoNS) [56]. The findings indicate that *C. sativa* can be targeted as a natural source for developing antibacterial drugs.

Like other antibiotics, a plant’s secondary metabolites encounter a barrier at the outer membrane of GNB, and limited efficacy is observed [108]. Nevertheless, many studies show *C. sativa* having a moderate to large inhibitory zone for Yersinia enterocolitica [79,83,107], *Vibrio cholerae* [73], Citrobacter freundii CCM 7187 [84], *Erwinia carotovora* [109], *Bordetella bronchioseptica*, *Proteus vulgaris* [105], *Aeromonas hydrophyla*, *Beneckea natriegens*, and *Flavobacterium suaveolens* [79].

It can be assumed that the bioactivity of *C. sativa* extracts and EOs fundamentally come from compounds such as cannabinoids, phenolics and terpenes [60,101,110]. The anntimicrobial profile of low-level THC content of *C. sativa* (industrial hemp) is partially related to CBD [94], CBDA [103], phenolics including flavonoids, caffeoyltyramine, cannabisin and polyphenols [99,101] and terpenes including α-pinene, α-humulene, β-pinene, β-caryophyllene, (E) caryophyllene, caryophyllene oxide and myrcene [60,93,94,102,110,111].

## 3. Antifungal Activity

Both superficial and systemic fungal infections have increased due to the emergence of many immunological dysfunctions in people [116]. The management of fungal infections suffers from the unavailability of drugs, toxicity, resistance and relapse of conditions [117]. Therefore, finding new antifungal drugs to combat fungal infections is a priority. In agreement with the set threshold by Kuete and Dabur to ascribe the antimicrobial and antifungal properties of plant juices [118,119], *C. sativa* extract, EO and their phytoconstituents possess significant activity against a number of pathogenic fungi and algae (Table 3).

*Candida albicans*, a prevalent opportunistic pathogenic fungus to humans, which is resistant to fluconazole, exhibited higher susceptibility to *C. sativa* extracts, EO and other compounds. Moreover, EO of *C. sativa* has a full synergistic effect with fluconazole, resulting in a 16-fold reduction of MIC against *Candida* spp. [60]. *C. albicans* is part of a natural microflora that forms asymptomatic colonies on the skin and inside the body and can proliferate if the host has an immunosuppressed condition and cause superficial mucosal and dermal infections [120,121]. Activity against *Candida* species [67,73,74,105,107] *Fusarium* spp. [68], *Candida neoformans* [73] and *Aspergillus* [68,105,122] are documented. Antifungal activity is cultivar-dependent [123] and also related to the active compounds’ chemical structures [75]. The findings indicate that more intensive study on the fungicidal activity of *C. sativa* phytoextracts is required for the treatment of fungal infections, especially for external use.

## 4. Antiviral Activity: Special Focus on SARS-CoV-2

**Figure 2:**
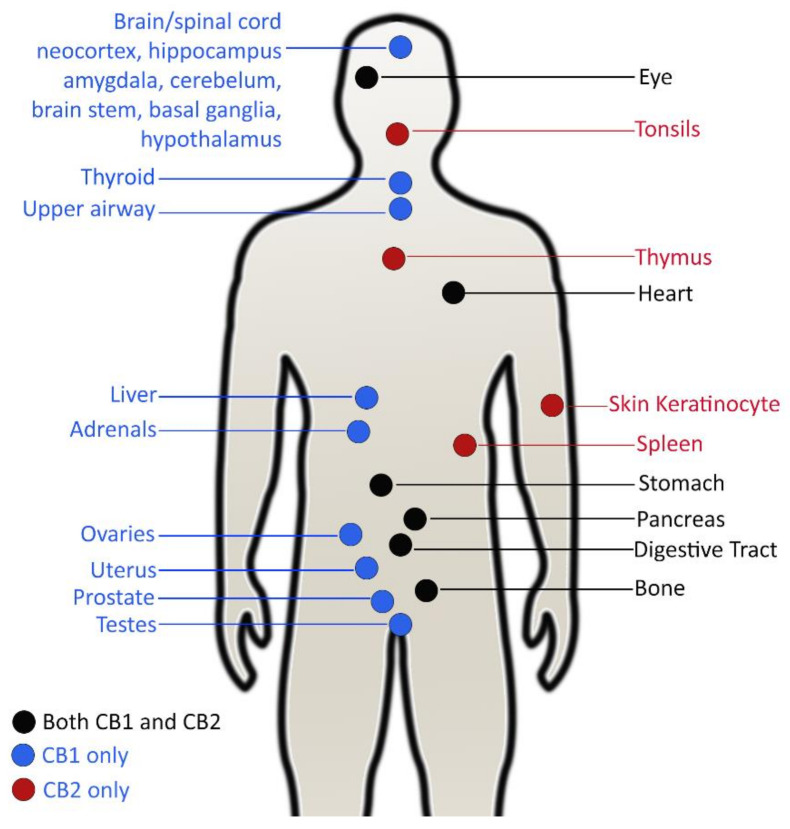
Location and distribution of main cannabinoids receptors in the human body (adapted from [129]).

Unlike bacteria, little literature exists on the direct antiviral activity of *C. sativa* compounds. Rather, they describe effects of cannabinoid receptors, CB1 and CB2 expressed in human cells through which cannabinoids assert their medicinal and psychoactive effects in viral infectious diseases. Psychoactive stimulation of cannabinoids is mediated by the CB1 receptor, which is largely expressed in the central nervous system (CNS), whereas the CB2 receptor takes part in immunomodulatory and inflammatory processes (Figure 2) [127]. The latter are predominantly expressed in tissues in the immune system and certain peripheral tissues [128].

Since the CB1 receptor is abundantly distributed in the CNS, its activation through cannabinoid agonists has considerable impacts on viral infections in neural tissues [130] and other places such as the lung [131] and liver tissues [132]. During viral infections, induction of CB1 receptors can activate some signaling pathways, reducing the concentration of cellular Ca^2+^ ions. As a consequence, it is likely to impair Ca^2+^-dependent enzyme release, nitric oxide production (NO), nitric oxide synthase (NOS 1) and pro-inflammatory mediators. These play important negative roles to boost host responses in viral infections and promote viral replication [29,130]. Activation of CB2 receptors expressed in immune cells has an influence on viral infections by altering immune response. The immunomodulatory and anti-inflammatory activity of CB2 receptors can cause reduction of the immune response, suppress inflammation, regulate cytokine production and mediate immune cells migration [27,127,133]. However, some results suggest that cannabinoids may exhibit pro-inflammatory activity in some cases [134].

The activation of cannabinoid receptors can regulate viral pathogenesis where host inflammatory and immune responses are associated with virus immunopathogenesis [29]. Their activation can reduce viral pathogenesis in many infections [27]; however, considerable contradictions exist in the literature, demonstrating the negative effects of cannabinoids in viral infections. For example, proliferation of viruses is a very common phenomenon in many infections [27]. Cannabinoid signaling can affect epigenetic changes that can inhibit the expression of genes related to cell-virus interaction and influence virus entry into the cell, virus replication and production, and host inflammation [27,135]. In vitro and in vivo studies showed that therapeutic use of immunoregulating cannabinoids led to disease progression, increases morbidity and even caused host death by suppressing antiviral immune responses [29]. Therefore, in certain viral infections, blocking of CB2 receptors is a drug target to inhibit immune-suppressive effects.

### 4.1. Antiviral Efficacy against Viral Pathogens

Several direct and indirect antiviral affects of cannabinoids in vitro and in vivo have been determined (Table 4). CB2 receptors activated by cannabinoid agonists are suppress the replication of human immunodeficiency virus (HIV)-1 [136,137]. THC (10 µM) exhibited a reduction of simian immunodeficiency virus (SIV) replication in MT4-R5 cells, which was administered 28 days prior to virus inoculation [138]. *C. sativa* plant extracts exhibited significant antiviral activity (*p* < 0.05) against Newcastle disease virus (NDV) [139]. THC inhibited lytic replication and reactivation of gamma herpesvirus, which indicates that ECS is possibly involved in regulating its latency. THC also showed inhibitory efficacy against the transcription factor ORF 50 gene promoter of murine gamma herpesvirus 68 (MHV) and Kaposi sarcoma-associated herpesvirus (KSHV) [140]. THC was found to have time, dose and concentration dependent suppressive effects on Herpes simplex virus (HSV), causing a significant decrease of virus viability [141,142]. Nevertheless exceptions occur as well [140].

An in vitro study of CBD uncovered its inhibitory property against hepatitis C virus (HCV) replication by 86.4% at a concentration of 10 μM [143]. In the infection of KSHV, CBD was found to inhibit the proliferation of infected human microvascular endothelial cells (HMVEC) while it had no impact on the ability to infect HMVECs when pretreated for 48 h. Dose-dependent CBD caused greater cell death (apoptosis) in infected cells than normal endothelium [144]. Among viral infections, psychoactive THC has been found advantageous from anecdotal and clinical evidence for the people suffering from acquired immunodeficiency syndrome (AIDS). The benefits do not come from virus inhibition or reduction of mortality but improvement in the host’s quality of life [145]. Dronabinol, a synthetic analog of THC, has been approved by the United States Food and Drug Administration (FDA) to mitigate wasting syndrome and loss of appetite of AIDS patients [146].

However, the anti-inflammatory activity of cannabinoids is the most attractive therapeutic potential, and may be crucial for many viral infections since it could mitigate the host immune response to pathogens [27,29]. The activation of cannabinoid receptors is beneficial in treatment when non-lytic viral infection is immunopathogenic [29]. The literatures indicated that evidence for the direct inhibitory effectiveness of *C. sativa* extract on viruses is limited; hence, more investigations with preclinical trials are required.

### 4.2. SARS-CoV-2

Severe acute respiratory syndrome coronavirus 2 (SARS-CoV-2), the latest member of the coronavirus group [147], has caused a devastating pandemic. Since effective therapeutics have still not been proven, and cannabis possesses tremendous effects on the human nervous system, as well as the human immune response, extracts of cannabis are included in the extensive search for new drugs against SARS-CoV-2.

**Figure 3:**
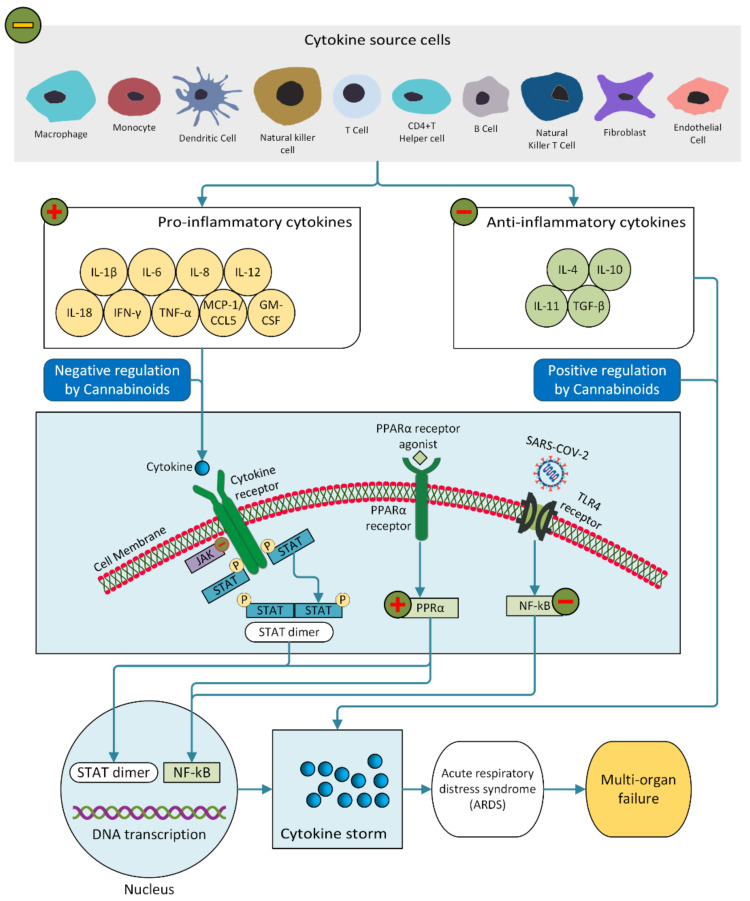
The impact of the cannabinoid system on the immune system in SARS-CoV-2 infection (adapted from [154]).

Evidence suggests that the severity and high mortality of COVID-19 are associated with a cytokine storm [25,148,149]. Pro-inflammatory cytokines IL-6, IL-8 and TNF-α are responsible for the cytokine storm in COVID-19 patients (Figure 3), leading to impairment of oxygen diffusion, pulmonary fibrosis and eventually multiple organ failure [150,151,152,153].

**Figure 4:**
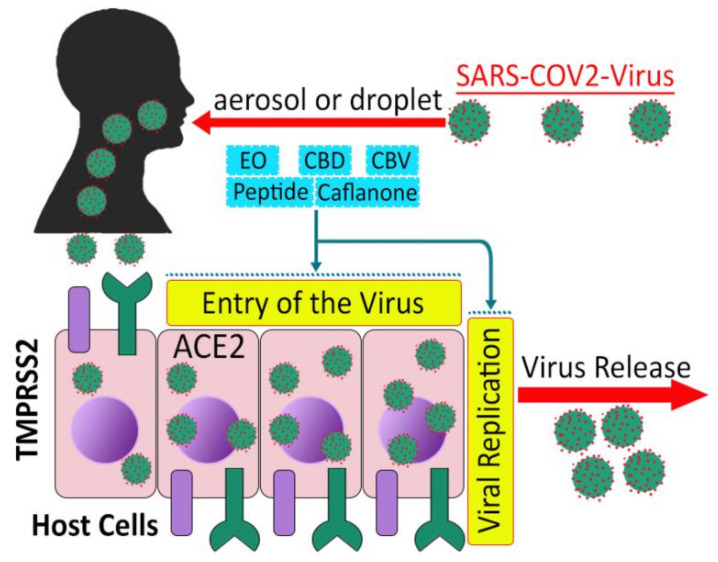
Potential effects of cannabis compounds on SARS-CoV-2 entry and replications (adapted from [160]).

Angiotensin-converting enzyme 2 (ACE2), a SARS-CoV receptor and transmembrane serine protease 2 (TMPRSS2), a cell surface protein, allow entry and S protein priming of SARS-CoV-2, respectively, thereby providing viral gateways (Figure 4) and viral activation in lung tissue, oral and nasal mucosa, and the gastrointestinal tract [155,156,157,158], and even facilitate mother to fetal transmission of the virus [159]. ACE2 enzyme binds glycoprotein S1, the characteristic viral spike protein, via a receptor-binding domain, and TMPRSS2 permits entrance of the virus into the cytoplasmic membrane of host cells [160,161]. Investigations also revealed that SARS-CoV-2 M^pro^ (the main protease) has a pivotal role in viral replication and transcription and can be regarded as an attractive drug target [162,163].

According to the latest report updated on 24 May 2021 by the National Institute of Health (NIH, USA), antiviral therapies could be more effective in the early course of COVID-19, while anti-inflammatory/immunosuppressive therapies are anticipated to be more effective in the last stage of disease [164]. Therefore, the study of cannabinoids as probable therapeutics during infection of SARS-CoV-2 has been reviewed, mainly illuminating their effects on inhibition of excessive cytokine secretion followed by anti-inflammatory amelioration [165,166,167,168,169,170,171]. A significant number of studies demonstrate that the anti-inflammatory activities of cannabinoids are characterized by a number of pathways such as the regulation of production, migration and functional ability of immune cells (macrophages, monocytes, neutrophils, lymphocytes, dendritic cells, killer cells, fibroblasts and endothelial cells), reduction of pro-inflammatory cytokines (IL-1β, IL-2, IL-6, IL-8, IL-12, IL-17, IL-18, IFN-γ, TNF-α, MCP-1/CCL5, GM-CSF) and increase of anti-inflammatory cytokines (IL-4, IL-10, IL-11, TGF-β) [21,154]. Since some contradictions exist in the literature [172,173,174], effects of cannabinoids on cytokine release are still arguable.

In lung injury, cannabinoids are found to be beneficial in vivo since they have a suppressive effect on the cytokine storm. Administration of CBD decreased migration of macrophages, neutrophils, and lymphocytes into the murine lung with acute lung injury (ALI) induced by LPS, followed by significant improvements of lung functions [19]. Crude and fractional extracts of cannabis reduced the level of IL-6, IL-8 in a lung epithelial A459 cell model [175]. Cannabinoid inhibited activation of NF-kB followed by a decrease of IL-8 release in HT-29 cells [176].

In clinical trials, tocilizumab, a recombinant humanized monoclonal antibody was used as an IL-6 receptor binder to assess the advantage of anti-inflammation in COVID-19 patients [150,177]. The results showed a statistically significant reduction of mortality and mechanical ventilator usage with a higher hospital release percentage [177]. A similar report involved administering IL-6 inhibiting monoclonal antibody sarilumab [178] and siltuximab [179] in separate clinical trials. Since siltuximab neutralizes IL-6, it has been approved by FDA for phase III clinical trials for hospitalized patients suffering from COVID-19-related acute respiratory distress syndrome (ARDS) [180]. Intranasal administration of CBD caused a reduction of pro-inflammatory cytokine IL-6 secretion, and symptoms were ameliorated by increasing apelin peptide in ARDS induced by simulated viral infection using poly (I:C) in mice [181,182].

Caflanone, a secondary metabolite from non-cannabinoid extracts of *C. sativa* [183] inhibited a number of entry factors for human corona virus hCov-OC43, pro-inflammatory cytokines (IL-1β, IL-6, IL-8, Mip-1α, TNF-α), ABL-2, cathepsin L, PI4Kiiβ and AXL-2 [184]. TNF-α was reduced by THC in a mouse model of Staphylococcal enterotoxin-B (SEB)-mediated ARDS [185] and in human cell line in vitro models [173]. The oral administration of THC and CBD in humans showed a significant reduction of TNF-α [186]. CBD combined with NT-VRL-1, an anti-inflammatory terpen formulation, exhibited potent antiviral activity against hCoV-E229E in the MRC-5 human lung cell line. It exerted additive or synergistic antiviral effects higher than that of the SARS-associated anti-coronaviral compounds pyrazofurin and glycyrrhizin [187]. Cannabis Science and Technology reports that an investigation is being undertaken by the Israel Institute of Technology to find a cannabis derived novel antiviral terpene formulation that could likely be effective against COVID-19 [188].

In vitro activity of THC and CBD against SARS-CoV-2 was determined (Table 5) following an interaction study involving an in silico experiment between 32 cannabinoids and SARS-CoV-2 M^pro^ [189]. CBD was characterized as a PPARγ agonist, so likely can exert antiviral activity and suppress the onset of the cytokine storm in COVID-19 infection, regulate fibroblast/myofibroblast activation and inhibit the development of pulmonary fibrosis, resulting in an amelioration of lung function in recovered patients [168]. Hemp EOs conferred significant inhibition of gene expression of ACE2 and TMPRSS2 in H1299 lung adenocarcinoma cell lines in basal conditions [123].

Computational studies such as docking scores, molecular dynamic (MD) simulation and density functional theory (DFT) showed that THC and CBD had stable conformations with the binding pocket of the M^pro^ enzyme [189]. The THC moiety and its derivatives have good stability and higher binding affinity in their complex with SARS-CoV-2 M^pro^ compared to a complex of hydroxychloroquine, remdesivir and their derivatives [190]. A HOMO-LUMO energy gap study also showed a good stability profile of THC and CBD with the SARS-CoV-2 M^pro^ enzyme [189]. It is notable that human proteases such as SARS-CoV-2 M^pro^ have not been reported with similar cleavage specificity, so inhibitors of this enzyme should not be toxic [163]. An in silico study revealed that caflanone has affinity to the viral spike protein, protease sites and helicase on the ACE2 receptor, and compared to chloroquine it showed higher binding energy with the spike protein, helicase and protease [184]. An MD simulation and docking study showed that the binding of CBD and cannabivarin (CBV) with ACE2, TMPRSS2, IL-6 and NRP1 (neuropilin 1) occurs, meaning that cannabinoids may be beneficial for CNS related post-COVID symptoms [191]. Neuropilin 1 is a protein which is encoded in humans (NRP1 gene), can interact with SARS-CoV-2 S protein and promote virus entry [192]. Since CBD-enriched extracts [155] and hemp seed extract’s active peptides [193] can downregulate ACE2 and TMPRSS2 enzymes, CBD is hypothesized for topical use as a preventive strategy against COVID-19 [168]. A number of drugs/drug classes are used clinically with various intake methods for other indications, such as ACE2 and TMPRSS2 inhibitors [161].

Finding efficient antiviral drugs against SARS-CoV-2 is of utmost concern for pharmaceutical scientists in this challenging time of the pandemic [194]. Though the present evidence is not enough for use of cannabinoids as pharmacotherapy against SARS-CoV-2, nevertheless, computational, in vitro and in vivo studies show selective cannabinoids and some non-cannabinoids have considerable effects on SARS-CoV-2 entry, replication, transcription, inhibition and significant anti-inflammatory effects that might have ameliorative effects in the host patients.

## 5. Mode of Action of Cannabinoids

The mechanism of antimicrobial activity of cannabinoids and extracts is still not established [85]. However, it is assumed that antimicrobial potentiality of plant extracts and EOs do not involve one solitary mechanism. Rather, many compounds and wide chemical profiling of extracts trigger several mechanisms at the cellular level to develop toxic activity against pathogens [196]. Although GPB possess a thicker peptidoglycan layer, they have a good response to particular cell wall-targeting antibacterial compounds, because they do not have an outer membrane [126]. The mechanism to inhibit GPB relies on invading the bacterial cell wall through cytoplasmic leakage and its coagulation [101]. CBD shuts down DNA, RNA and peptidoglycan synthesis of *S. aureus* and penetrates biofilms [49]. CBCA invades the structural integrity of the bacterial lipid membrane, alters bacterial nucleoids, and causes bacterial cell death [52].

A docking study indicated that the penicillin-binding proteins (PBP) of *S. aureus* could be a target for cannabinoids since the most active cannabinoids have greater affinity to PBP, whereas less active compounds show less affinity. Moreover, lower polarity and lipophilicity of cannabinoids could enhance the probability of attacking bacterial membrane proteins [197]. Chemical genomic profiling of MRSA with sublethal concentrations of CBG indicated that the activity of CBG was linked with impairment of the cytoplasmic membrane [43]. The cytoplasmic membrane has a critical role in cell functioning and survival for both persisting and non-growing cells [198]. EO at sublethal concentrations weakens the biofilms invading Caco-2 cells of *L. monocytogenes* and significantly induces their motility [111].

The lower susceptibility of GNB to antibacterial agents is attributed to the barrier created by the outer membrane hydrophilic LPS of the bacteria, which acts as a wall to macromolecules and hydrophobic antibacterial compounds present in the extracts [78,199,200,201]. The intensity of action of extracts on GNB depends on the extent of disturbance and inactivation of the function of the outer membrane by abandoning LPS [196].

The activity of CBD against GNB biofilms may be through the disruption of membranes [49]. It can change the protein profiles of MVs released from *E. coli* and possesses strong inhibitory efficacy due to that [63]. CBG itself alone is unable to kill GNB, but an addition of less toxic polymyxin B nonapeptide, a derivative of polymyxin B, can perturb the outer membrane to allow the access of CBG into the cell, and eventually it reaches the inner cytoplasmic membrane and disrupts its integrity [43]. CBD attacks N. gonorrhoeae and Moraxella catarrhalis, whereas LPS is not an essential outer membrane building block [49]. The presence of naringenin in EO affects modification of cell membrane fluidity in *S. aureus*, induces bacterial genes related to fatty acid biosynthesis and modifies fatty acid composition [61].

The inhibition of plasmid transfer and transformation by THCA and cannabispiro compounds may be characterized by restricting mating pair formation, zygote growth, trans-conjugal DNA synthesis, DNA penetration, and the synthesis of plasmid DNA during cell growth [88]. As a whole, the antibacterial efficacy of EOs and extracts has been shown to involve damaging the cytoplasmic membrane, ion leakage, loss of energy sources such as glucose and ATP, and coagulation of cell contents by inhibiting the production of amylase and protease [117,202]. All these inevitably cause lysis of bacteria and bacterial death.

Locating the antifungal mechanism of plant extracts and EOs is difficult, due to lack of definite insights into antifungal factors in the extracts that have effects against pathogenic fungi [196]. The exploration of active compounds in plant extracts with substantial antifungal activity is, therefore, required to fight against drug-resistant fungi. Antifungal attributes may be ascribed to polyphenolic compounds and oxygenated monoterpenes [117], and they exert similar modes of action to those of bacteria, including irreversible impairment of the cell septum, oozing and coagulation of cellular materials [196], but additionally, producing a pH gradient across the cytoplasmic membrane and preventing energy production of yeasts are worth mentioning [202].

A large variety of antiviral phytochemicals from hundreds of plants have been identified with overlapping and complementary mechanisms of action [203]. β-caryophyllene, a terpene compound present in *C. sativa* and many other EOs, is widely claimed to have antiviral activity. EOs, as blends of myriad metabolites, inhibit viral nucleic acid (DNA/RNA) synthesis and alter structural proteins to arrest the virucidal effect and inhibit specific processes in the viral replication cycle that prevents cell-to-cell virus diffusion [202,203].

## 6. Factors Affecting Antimicrobial and Antiviral Activities of Phytocompounds, EOs and Extracts

### 6.1. Physical Factors

Bioactivity of *C. sativa* EOs and extracts depend on the concentration of active compounds, which is associated with many extrinsic factors such as geographical origin, sowing time, plant age, collection time, and soil composition, along with many intrinsic factors including genetic information, cultivars, accessions, maturity, and even the aging of the extract itself [60,79,83,204]. Appropriate selections of solvents, their concentration, extraction method and extraction parameters are crucial for biocidal property of respective extracts [74,205]. Apart from these, the choice of antimicrobial tests also produces variability in results [206,207]. Antibacterial activity significantly differs with biomass, distillation time, and interaction between material and distillation time [107]. This happens because the composition of EOs strictly depends on distillation condition and the state of the plant when distillation occurred [208]. EOs from unground plant material with a low distillation time has more antimicrobial activity than EO from ground plants material with a long distillation time. In cannabis resin, an unripe sample contains more CBDA; hence more activity occurs [103]. Wild hemp exhibited significantly more antimicrobial efficacy than registered cultivars [83]. EOs from Futura showed minimum MIC against GPB compared to Carmagnola and Fibranova [93]. Organic extracts showed greater activity than aqueous extracts [62,68,81], and stem parts had more traits than leaf or root parts [62,68,81]. Extraction of active compounds from fibers reduces their antimicrobial activity, and the reduction depends on extraction time [209]. The antibacterial traits of hemp hurd powder are associated with retting and microbial contamination and are independent of particle size. The activity is enhanced by heat treatment of an appropriate time and temperature [210].

### 6.2. Structure

The activity of cannabinoids is supposed to be linked with the presence of the prenyl moiety and its relative position [95]. The resorcinol moiety of cannabinoids serves as the antibacterial pharmacophore, with the alkyl, terpenoid, and carboxylic appendices modulating its activity. Bactericidal activity against MRSA is neutral to the nature of the prenyl moiety, to its relative position compared to the n-pentyl moiety (abnormal cannabinoids), and to carboxylation of the resorcinyl moiety (pre-cannabinoids). The introduction of a second prenyl moiety, methylation and acetylation of the phenolic groups, and esterification of the carboxylic group of pre-cannabinoids are detrimental to the antibacterial activity of all five major cannabinoids [95]. In another study, modification by replacement of n-pentyl with n-propyl and benzoic acid moiety decreased the susceptibility of MRSA to all major cannabinoids [43].

Maximum activity is manifested for CBC type compounds when a methyl or pentyl group is held in the side chain [75]. The activity is enhanced for CBC and CBG-type compounds having a methyl side chain due to saturation of two double bonds in the compounds. The compounds holding hydrogen instead of R (hydrocarbon or hydroxyl group) in the chemical structure show medium activity, whereas it is reduced with lengthening of the side chain [75]. According to Turner and coworkers, the n-pentyl chain meta to the alcohol group of CBC analogs seems to be vital for activity against *B. subtilis* and *S. aureus* [211]. The action of CBD is maintained with alteration of its core structure, which indicates that systematic activity with reduced protein binding profile can be achieved by modification of physicochemical properties [49].

The activity against Gram-positive and GNB is assumed to be linked to the phenolic hydroxyl groups forming hydrogen bonds of caffeoyltyramine present in seed extracts with active sites of target enzymes [101]. New structures of cannabinoids coupled with an oxacillin beta-lactam ring have been proposed based on a quantitative structure-activity relationship (QSAR) study. The interaction between the hydroxyl group of aromatic rings of cannabinoids and PBP of bacteria resulted in lower MIC and better drug-likeness scores against MRSA [197]. In the case of endocannabinoids, the fatty acid structure of EC and EC-like compounds is supposed to determine their activity against *S. mutans* [212]. Acidic conditions and metal ions can modulate the activity of fractioned extracts. It was reported that acidic conditions, as well as Ca^2+^, K^+^ and Na^+^ increase activity against *S. aureus* and Listeria, whereas Fe^3+^ reduces it [213].

### 6.3. Synergism

All major and minor antibacterial components in EOs are important contributors due to synergism, because sometimes a major component may exhibit less activity than a mixture of components [94,107,214]. The activity of pure THC, CBD and a 1:1 mixture displayed marginal antibacterial activity compared to raw extracts [108]. A greater inhibitory zone was found for unrefined EOs compared to refined oil [84]. Therefore, it is difficult to correlate the relationship between the amounts of active compounds in EOs and their bioactivity. This strongly suggests that synergism occurs in EOs, which causes enhanced penetration of active molecules into the bacterial cells [94]. A combination of cannabinoids to terpenes at a 5:1 ratio resulted in a maximum zone of inhibitions against Gram-positive and negative bacteria [77]. Efficacy was reported to increase for cannabis-dominated ginger extracts [126].

## 7. Potential Application of Antimicrobial Properties of Cannabinoids in Non-Drug Agents

### 7.1. Cosmetics (Toothpaste)

The effects of cannabinoids on bacterial growth in dental plaque have been investigated in recent years [215,216,217]. Dental plaque, a complex biofilm, acts as a reservoir of microbes that can initiate several dental problems. Cannabinoid-infused mouthwash (1% CBC and CBG) had the same bactericidal efficacy as 0.2% chlorhexidine [215]. The chlorhexidine-containing mouthwash has been reported as most effective in controlling dental plaque, but it causes tooth staining [218]. Therefore, cannabinoid-infused mouthwash has interest as a safer and efficient alternative. Diethyl ether and acetone leaf extract exhibited an MIC value of 5.0 and 2.5 mg/mL, respectively, against dental microflora [216]. Several formulations containing 12.5% major cannabinoids (except psychoactive Δ^9^-THC) exhibited more effectiveness in reducing colony forming units than those of popular oral care products such as Oral B and Colgate [217]. All the above can lead to new formulations of toothpaste without side effects.

### 7.2. Food Plants

Food-borne pathogens cause millions of illnesses every year, representing one of the most vital public health problems worldwide [219]. Microorganisms form biofilms on food contact surfaces such as stainless steel in food plants, and if not disinfected well, these pose a constant threat of contamination in foods, food packages, and instruments which can cause illness in processing personnel and consumers [220]. To eradicate microbial contaminants, food plants practice traditional techniques and physical and chemical methods [220], which have led to development of resistance to disinfectants in many pathogens [221]. Since plant extracts are often antimicrobial and have many synergies with synthetic antibacterial agents, they have received attention by researchers for their sanitization activity in food plants [78,222]. Activity of *C. sativa* extracts has been detected for many food-borne pathogens (Table 2) including *S. aureus*, *E. coli*, *L. monocytogenes*, *K. pneumoniae*, *H. pylori*, *S. typhimurium*, *Y. enterocolitica*, *B. cereus*, *Shigella* species, *Aeromonas* species and so on. In this regard, *C. sativa* should be interesting in drug design for food-borne illness and as a disinfecting agent for food plants.

The survival ability of *L. monocytogenes* biofilms and resistance to biocides, including sanitizer/disinfectant, increases complications in food processing plants [223]. *L. monocytogenes* causes listeriosis in humans and animals and exhibits resistance to broad-spectrum of cephalosporin antibiotics [224].

Contamination in food plants caused by MRSA biofilms on solid surfaces is another serious issue for public health [225]. Hemp seed extract has potential as an antibacterial agent in food plants to fight MRSA biofilms because it can inhibit virulent biofilms at low concentration [101]. Hemp EO can impede the formation of *S. aureus* biofilms and planktonic cells without affecting the growth of probiotic strains belonging to the *Bifidobacterium* and *Lactobacillus* [101]. An ethyl acetate fraction of leaf extract was ascribed for good efficacy against *S. aureus* and *L. monocytogenes*, which were unaffected by different temperature treatments, sucrose addition and ultraviolet irradiation [213].

Among Gram-negatives pathogens, *Pseudomonas* [93], *Shigella* [76,82], *Salmonella enterica* subsp. Enterica, *Salmonella typhi* [76,80,83,107] and *Y. enterocolitica* [83] exhibit good sensitivity to hemp EOs or solvent extracts. EOs and terpenes are remarkably effective against a broad range of Gram-positive and Gram-negative food-borne and spoilage organisms (Table 2) [93,97,108]. *Torulospora delbrueckii* and *Zygosaccharomyces bailii* are two spoilage-causing yeasts in food and beverages including soft drinks, fruit juices, vegetables, meats, salads and dairy product [226,227] and have significant susceptibility to EOs and terpene compounds from a variety of industrial hemp, with MIC 0.91–1.94 (%*v*/*v*) [93]. Addition of CBD (6.45 µg/gm) in minced beef could reduce *Enterobacteriaceae* and coliform counts and was found to inhibit spoilage bacteria belonging to the strict aerobic *Pseudomonas* species [228]. The use of *C. sativa* extracts in food plants as antibacterial agents must have low THC content from certified industrial hemp, rather than an unknown variety, because the use of THC in consumer products is strictly regulated in some countries. However, more toxicity studies are required before the full-scale application of cannabis EOs or extracts as disinfectant/sanitizing/anti-spoilage/food preservative agents in food plants.

### 7.3. Crop Protection

Plant diseases caused by pathogens have crucial impacts on food security and the economy in every country in the world. The most common and dominant plant pathogens belong to fungi [229,230]. Fungal plant pathogens cause not only yield loss but also deteriorate the quality of field crops and edible plant parts [231]. The utilization of synthetic fungicidal agents is a general practice, and some agents leave residues and cause soil and water pollution with serious ecological impacts [232,233]. In this regard finding less toxic, ecofriendly natural resource-based agents is of growing interest. *C. sativa* EOs, solvent extracts and their many individual compounds have considerable antifungal attributes against both human and plant pathogens.

A 15% leaf extract of *C. sativa* had 100% inhibition of mycelial growth of *Curvularia lunata* [234]. *C. lunata* causes leaf spot [235], leaf blight [236], stem blight [237] and root rot [238] in a variety of agricultural crops. The extracts had concentration-dependent antifungal properties against *Sclerotium* (*Athelia*) *rolfsii* [239], *Fusarium* spp. [68], *Cryptococcus neoformans* [73] and *Alternaria* species [234,240,241]. *Alternaria* species cause a range of plant diseases in many agronomic host plants including oil crops, cereals, ornamentals, vegetables such as potato, broccoli, cauliflower, and carrots, and fruits such as apple, tomato and citrus. Furthermore, *Alternaria* spp. are regarded as post harvesting pathogens [242].

Terpene compounds had higher activity with minimum MIC 0.091 (%*v*/*v*) than EOs of the inflorescence from industrial hemp against a panel of phytopathogens including *Pichia membranaefaciens*, *Saccharomyces cerevisiae*, *Kluyveromyces marxianus*, [93]. EOs of industrial hemp cultivars and their terpene compounds showed good activity (MIC 1.24–1.84 %*v*/*v*) against plant pathogenic bacteria including *Pectobacterium* [93]. *Pectobacterium* species cause soft rot, stem rot and blackleg in potato, and in a wide range of other vegetable crops and decorative plants [243]. Integrated Pest Management (IPM) strategy may be undertaken for extracts with inadequate fungicidal activity by combining with synthetic compounds in order to reduce negative environmental effects [234].

### 7.4. Others Application

Since the antimicrobial characteristics of *C. sativa* extracts are well documented, their application in functional materials where microbial infestation is a concern is anticipated. Ultrafiltration hybrid membranes made of surface-modified Polyethersulfone with a mixture of cannabinoids/terpenes (5:1) showed outstanding performance against the proliferation of pathogenic Gram-positive and negative bacteria without compromising functionality [77]. A green biocidal finishing agent for textile applications produced from extracts of hemp fiber has been invented [244].

The antimicrobial efficacy of hemp fiber and hurd has been demonstrated too. Hemp fiber possesses antimicrobial traits against *C. albicans*, *S. aureus*, and *E. coli* [209], whereas hurd is active against *E. coli* [210]. The solid fiber of *C. sativa* had 85% inhibition of mycelium growth against *C. albicans* [209]. A chelating biopolymer has been designed using hemp fiber and a biocidal agent to remove metal ions from aqueous solutions and showed inhibitory performance against *S. aureus* and *P. aeruginosa* [245]. A surgical device made from antibacterial hemp fiber has been patented [246]. The presence of antibacterial β-sitosterol, β-amyrin, alkaloids, flavones, saponins have been suggested for antimicrobial attributes in hemp fiber [209,247]. On the other hand, hemp hurd contains a high amount of lignin, and lignin-related compounds including phenolics, alkaloids and cannabinoids may be involved in hurd’s antimicrobial features [247,248].

## 8. Challenge vs. Opportunity as a Pharmaceutical Drug

The historical evidence of medicinal use of cannabis has been in the ancient Chinese Pharmacopoea, Shen Nung Pen Ts’ao Ching, written in the first century before the current era [249], and has remained in British Pharmacopeia since 1932 [250]. Although the UK prohibited its medical use in 1973, in the first century of the current era, Britain used cannabis as a mainstream medicine to alleviate pain, fever, insomnia, convulsions, muscle spasm, prolonged labor, nausea, migraine, dysmenorrhea and asthma [250]. The research on the medical use of cannabis has escalated since its pharmacological and toxic properties, along with cannabinoid structure, were revealed in the nineteenth century [249]. In 1851, cannabis was included in the third edition of the United States Pharmacopeia for use of its flower as an analgesic, anticonvulsant, and hypnotic, but in the 12th edition it was removed in 1941 [249]. The controversy surrounding the medicinal use of cannabis is still a subject of debate [251]. Some countries have legalized medical cannabis, but it is not yet considered a pharmaceutical drug because of fear and stigma, lack of standardization and legalization without standard critical trials [26].

Studies have revealed that, unlike synthetic drugs, the therapeutic advantage of cannabis is attributed to combined mechanisms of blended compounds as the result of synergisms or antagonisms [252]. Synergy may occur among cannabinoids (intra-entourage) or between cannabinoids and terpenes (inter-entourage) [54,253]. Terpenes and flavonoids play essential roles in modulating cannabinoid functional ability by altering pharmacokinetics and permeability [249]. After all, the individual compounds have their own pharmacology, too [250]. They can either increase therapeutic activity or decrease toxicity by interacting with many cellular and physiological systems in the body [249,254]. The main controversy arises regarding medicinal use of cannabis due to toxicity of some cannabinoids, especially THC, which is found in dried inflorescences from female plants known as marijuana [249] and is a widely abused recreational drug [255]. Whether it is taken by smoke inhalation or ingested, the toxicity principally links to the CNS, respiratory and endocrine systems. It has pivotal psychotropic effects including exhilaration, hallucinations, delusions, blurred vision, poor coordination, stupor and coma [256]. There is also evidence that THC accumulates in the brain [257]. For these reasons, psychoactive cannabinoids at higher doses are not used for clinical applications. In this regard, non-psychoactive cannabinoids such as CBD and CBG are promising. In contrast to THC, they possess ki values (inhibitor constant) greater than 2300 nM and have less affinity to CB receptors [258]. Lethal doses are much lower for THC (LD_50_ > 100) on mice than its analogs, CBD and CBD’s analogs [259]. Escalated doses of THC (up to 49 mg/kg) and CBD (up to 62 mg/kg) are safe, with mild adverse effects on dog [260]. CBD showed modest cytotoxicity against HEK-293 cells and did not show signs of hemolysis up to 256 µg/mL when exposed to human blood cells [49]. CBD, CBG, Δ^9^-THCV and CBDV resulted in rapid penetration of the blood-brain barrier after a single-dose (120 mg/kg, 120 mg/kg, 30 mg/kg, 60 mg/kg, respectively) via intraperitoneal and oral administration in mice and rats without revealing acute symptoms of toxicity [261]. A dose of 100 mg/kg of CBG was found most effective, without significant change of mouse weight, over various time points [43]. CBG and CBGA did not show any cytotoxic effects on African green monkey kidney fibroblast Vero cells [114].

In the light of the toxicity of EO and extracts at varied doses, EO of Nepalese hemp had a lethal concentration (LC_50_) 13.6 µg/mL to brine shrimp, and this was >200 µg/mL for nematodes, worm larvae, insecticides and flies [262]. An aqueous extract of industrial hemp did not show toxicity on brine shrimp at concentrations ranging from 0.1–20 mg/mL (LC_50_ 1.156–2.696 mg/mL). The extracts remained ineffective at a concentration of 100 µg/mL in HCT116 cells in modifying cell migration, which might suggest the dose as a good biocompatibility limit for pharmacological evaluations [99]. In another study with an in vitro model constituted by human H1299 lung adenocarcinoma cells, EO of industrial hemp at 0.0625–0.25 µL/mL did not have effects on cell survival in basal conditions [123]. Hemp EO showed inhibition of cell viability in some cancer cells such as Caco-2, Mz-ChA-1, MCF7 and MDA-MB-468 cells with IC_50_ values of 28.7, 22.3, 83.2 and 53 µg/mL. Cell proliferation was inhibited by 44% using EO (250 µg/mL) in nonmalignant cholangiocytes (H69) [61]. The lethal dose (LD_50_) of EO was recorded at 1.56 mg/mL on larvae of *Galleria mellonella* [61]. In the screening of hemolytic activity, hemp extracts showed 1.97 to 5.88% lysis of RBC against human erythrocytes [62]. An introduction of 4 and 8 mg ethyl acetate seed and leaf extract showed toxicity to 9-days old chicken embryos [139]. For more detailed information on dose vs toxicity of cannabis, readers are referred to [19,263,264,265].

To be a drug candidate, not only safety is an issue but compounds also need to achieve drug-like properties such as solubility, permeability, metabolic stability and transporter effects (influx and efflux) [266]. The overall structural properties, physicochemical properties, biochemical properties, pharmacokinetics and safety profile with regard to the pharmacology of individual antimicrobial compounds and mixtures need to be critically analyzed [252,267]. Cannabinoids have challenging pharmacological properties, and their pharmacokinetics depend on the route of administration, dosing, formulation and preparation of a certain product [254,268]. There is evidence that the onset, rate of absorption and bioavailability of CBD and THC are significantly lower after ingestion or oral administration than after inhalation [254]. The activity of THC and CBD against GPB in media containing 5% horse blood and 4% serum was recorded to be very poor: likely, binding protein in the media and quickly disappearing from the blood [115]. Similar evidence was reported for CBD with 50% human serum [49], which means the compounds lack systemic activity and have complexity when used as a therapeutic [115].

CBG exhibits several desirable physicochemical properties in terms of molecular weight, number of rings and rotatable bonds, and the number of hydrogen donors and acceptors, but suffers from higher lipophilicity and low aqueous solubility [43]. Classical phytocannabinoids are soluble in lipids and nonpolar organic solvents [269]. Lipophilicity ranges between LogP 4.96 to 8.59, and can be ranked as follow: CBG > CBC > CBT > CBD > CBE > THC > CBDV > CBN > CBL [270]. A LogP less than 5 indicates better ligand bioavailability [197]. Higher lipophilicity allows cannabinoids to cross the blood-brain barrier and be readily distributed to lipid-laden tissues and neuronal cell membranes [271]. The synergism of phytochemicals present in cannabis also leads to increased bioavailability and penetration through the blood-brain barrier [249].

Resistance propensity to target bacteria is a critical parameter for any new antibiotic. So far it has been assessed for CBD [49] and CBG [43]. CBD showed a lower innate resistance frequency value against MRSA, and its MIC increased only 1.5-fold over 20 days of daily passage [49]. The rate was also found to be lower for CBG at a subconcentration of MIC. The safety profile and low resistance propensity of compounds provide an important indication for new antibiotics [49]. The structure-activity relationship of CBD is similar to prototypical narrow spectrum antibiotics and has the potential to develop new analogs against Gram-negative *N. gonorrhoeae* [49]. CBD and CBDA displayed a wide gap of concentrations between hemolytic activity and MIC in human erythrocytes which indicates that the compounds have significant interest for new drug development related to blood conditions [50].

In the investigation of drug-like properties of phytocannabinoids by an in silico study [272], compounds including CBD, CBDA, CBC, CBG, CBN, THC, and many others, were found to have molecular weights of <500, a number of hydrogen acceptors (HBA) <10, a number of hydrogen donors (HBD) <5, a topological polar surface area (TPSA) <140 Å^2^ and a number of rotatable bonds (NRTOB) <10. They were found to have moderate to active bioactivity scores, except for CBDA and CBT, and all showed good oral absorption with a 100% absorptivity. Cannabitriol (CBT) does not violate any of Lipinski’s rule of five, whereas all other tested cannabinoids have one violation, indicating that the compounds have good bioavailability. Tetrahydrocannabivarin (Δ^9^-THCV), CBDA, cannabicyclol (CBL), cannabielsoin (CBE), and CBT have active drug-likeness scores of 0.07, 0.20, 0.20, 0.39 and 0.57, respectively. A QSAR study proposed three predicted modified structures of cannabinoids having better drug-like properties with a LogP less than 5 [197]. In a tetrad test, cannabinoids had the same pharmacological properties as other centrally acting drugs in vivo [273]. QSAR, drug-likeness and docking properties of cannabinoids and their modified structures elucidated the probabilities of their effectiveness against MRSA strains [197]. Recently, frontier orbitals (HOMO-LUMO) of compounds have been of importance in regulating many biological activities, including antibacterial and antifungal effects. A study revealed the similarity of frontier orbital distribution for three cannabinoids to commercial antibiotics, elucidating that those might be considered as the most potent pharmaceutical compounds [197].

Extracts are sometimes suggested for topical use for treatment of skin disorders caused by biofilms of antibiotic resistant bacteria [95,102,115]. Based on the anti-inflammatory and antimicrobial properties of CBD, phase II clinical trials are undergoing for the topical treatment of acne (NCT03573518) and atopic dermatitis (NCT03824405) [49] and for nasal decolonization of MRSA [274]. In a study, the killing ability of CBD in a topical application was found to be highly formulation-dependent, and a high level of CBD was not effective unless delivered in a compatible vehicle [49].

It is fortunate that several orally administered synthetic and plant-derived drugs have been approved by regulatory bodies in some countries. Dronabinol (a synthetic form of Δ^9^-THC) was approved by the FDA in 1985 for the treatment of anorexia associated with weight loss in adult patients with AIDS, and nausea and vomiting associated with cancer chemotherapy where conventional antiemetic treatments failed [250,275]. Another synthetic, but structurally distinct derivative of Δ^9^-THC, nabilone, was licensed in the UK, Canada and USA for the treatment of nausea and vomiting caused by chemotherapy when it is unresponsive to conventional antiemetics [250,275]. The synthetic compound does not interact with other compounds, as observed with the combination of phytochemicals [249].

From a natural source, Epidiolex, a 98% pure cannabis-derived oral CBD solution, was recently approved by the FDA for the treatment of epileptic seizures associated with Lennox-Gastaut syndrome (LGS) and Dravet syndrome (DS) in pediatric patients from 2 years of age and older. Since it has no harmful effects, the drug is under consideration for use in inflammation, cancer, neurodegenerative diseases and diabetes [275]. However, purification of compounds from the crude extract is a challenge [194]. Nabiximols, an oral spray containing plant-derived purified THC, CBD and other minor cannabinoids and terpenes, is legal for medical use in more than 25 countries other than the USA for the treatment of painful spasticity and neuropathic pain in multiple sclerosis. In the USA it is an investigational drug for advanced cancer pain, polyneuropathy, HIV-associated neuropathy and palliative care [275]. Dronabinol, nabilone, nabiximols are reported to have adverse effects relating to the CNS, cardiovascular and respiratory systems [276]. Moreover, evidence exists that cannabis has pivotal impacts on infectious diseases [277]. Recently, the FDA granted a synthetic cannabinoid based antimicrobial product, BTX 1801, Qualified Infectious Disease Product (QIDP) designation status. QIDP is an FDA program designed to provide incentives for the development of novel antibacterial and antifungal products [278].

## 9. Conclusions

*C. sativa* is considered one of the most controversial plants in our society but, at the same time, it has been used worldwide for medicinal purposes for centuries. Since the plant kingdom is now drawing a considerable interest for new antimicrobial and antiviral drugs, and *C. sativa* has great interest as a medicine, its proven antimicrobial efficacy is emerging as new therapeutic candidate or prophylaxis measure in fighting antibiotic resistance and COVID-19. The anti-inflammatory effects of cannabinoids are well-proven and already being used for other indications. Therefore, their impacts on COVID-19 need to be investigated extensively. Computational studies with regard to the SARS-CoV-2 main protease are interesting, as is searching for its efficacy in depth. However, this will lead to new pharmaceuticals only if the new drug can target specific pathogens or receptors with sufficient efficacy in infectious diseases without showing any objectionable toxicity. Therefore, important pharmacological profiles, including absorption, distribution, metabolism, mode of action and elimination, versus toxicity of individual cannabis component and their complex mixtures with specific antibiotics, need to be defined accurately. More in vivo studies and preclinical trials are required with a large number of participants. Besides, natural antimicrobial cannabis products have potential to be used in food industries and agricultural pesticides. However, any cannabis products made into antimicrobial agents must satisfy strict requirements by regulatory bodies in terms of quality, safety, efficacy and cost effectiveness, following good laboratory practice, good manufacturing practice and good clinical/ application practice.

## Figures and Tables

**Table 1:** Activity of cannabinoids and *C. sativa* against the resistant pathogens enlisted in WHO’s current priority list.

Pathogen	Compound/Extract/EO	Activity	Reference Antibiotic		Ref
			Antibiotic	Activity	
***Gram*** **+*ve***					
*Enterococcus faecium*	EO, α-humulene, α-pinene, β-pinene, myrcene	MIC 0.75–1.87 (%*v*/*v*) MBC 1.39–2.83 (%*v*/*v*)			[93]
*E. faecium*	EO, α-humulene, α-pinene, β-pinene, myrcene	MIC 1–4 µg/mL	Ciprofloxacin	MIC 8 µg/mL	[94]
EMRSA 15 and EMRSA 16	CBD, THC, CBG, CBC, CBN	MIC 0.5–2.0 µg/mL			[95]
MRSA	4-acetoxy-2-geranyl-5-hydroxy-3-n-pentylphenol and 8-hydroxycannabinolic acid A	IC_50_ 6.7 µM	Ciprofloxacin	IC_50_ 0.4 µM	[96]
MRSA	CVDVM	MIC 15.6 µM			[52]
MRSA	CBCA	MIC 3.9 µM			[52]
MRSA	CBD	MIC 1 µg/mL	Tobramycin, Meropenem, Ofloxacin	MIC 1, 16, 64 µg/mL (respectively)	[50]
MRSA	CBD	MBEC 2–4 µg/mL			[49]
MRSA	CBD analogs	MIC 0.25–64.0 µg/mL	Vancomycin, Daptomycin, Mupirocin	MIC 0.125–2.0 µg/mL	[49]
MRSA	CBD, CBN, CBC, CBDV and Δ^1 & 9^-THC	IC_50_ 5.8–10.6 µM	Ciprofloxacin	IC_50_ 9.33 µM	[97]
MRSA	CBDA	MIC 4 µg/mL	Tobramycin, Meropenem, Ofloxacin	MIC 1, 16, 64 µg/mL (respectively)	[50]
MRSA	CBG	MIC 2 µg/mL and MBEC 4 µg/mL			[43]
MRSA	EO	IC_50_ 0.82–4.22 µg/mL			[98]
MRSA, VISA, VRSA, *E. faecium*	CBD	MIC 1–2 µg/mL	Vancomycin, Daptomycin, Trimethoprim, Mupirocin, Clindamycin	MIC 0.125 to >64 µg/mL	[49]
*Streptococcus pneumoniae*	CBD	MIC 1–4 µg/mL	Vancomycin, Daptomycin, Trimethoprim, Mupirocin, Clindamycin	MIC 0.25 to >64 µg/mL	[49]
VRE	CBCA	MIC 7.8 µM			[52]
**Gram -ve**					
Escherichia *coli*	Aqueous extract	MIC 7.14 mg/mL	Ciprofloxacin	MIC < 0.12 mg/mL	[99]
*E. coli*	N-*p*-*trans*-coumaroyl-tyramine	IC_50_ 0.8 µg/mL	Ciprofloxacin	IC_50_ 0.01 µg/mL	[100]
*E. coli*	Seed extract	MIC 25 µg/mL			[67]
*E. coli* and *Salmonella typhimurium*	Seed extract	Growth inhibition at 1 mg/mL			[101]
*E. coli*, and *Pseudomonas aeruginosa*	EO	MIC 1.2 mg/mL		MIC 0.062–1.0 mg/mL	[60]
*Enterobacter aerogenes*	Seed extract	MIC 2.5 mg/mL			[101]
*Neisseria gonorrhoeae*	CBD	MIC 1–2 µg/mL	Vancomycin, Levofloxacin, Meropenem, Gentamicin	MIC 0.002–4.0 µg/mL	[49]
*N. gonorrhoeae*	CBD analogs	MIC 0.03–16.0 µg/mL	Mupirocin Colistin	MIC 1–32 µg/mL	[49]
*P. aeruginosa*	Aqueous extract	MIC 7.14 mg/mL	Ciprofloxacin	MIC 1.23 mg/mL	[99]
*P. aeruginosa*	Whole plant extract	MIC 12.5 µg/mL			[67]

**Table 2:** Activity of cannabinoids and *C. sativa* against pathogens other than those on the WHO’s priority list (* collected from foods or food environments).

Pathogen	Compound/Extract/EO	Activity	Reference Antibiotic		Ref
			Antibiotic	Activity	
***Gram*** **+*ve***					
*Bacillus subtilis* and *Staphylococcus aureus*	Leaf extract	MIC 1.56 mg/mL			[81]
*B. subtilis*, *S. aureus* and *Micrococcus luteus*	EO	MIC 1.2–4.7 mg/mL	Ciprofloxacin	MIC 0.015–0.031 mg/mL	[60]
*B. subtilis*, *S. aureus*, *Mycobacterium smegmatis*	CBC, its homologs and isomers	MIC 0.39–3.12 µg/mL			[112]
*Clostridium* species *, Enterococcus hirae *, Streptococcus salivarius *	EO, α-humulene, α-pinene, β-pinene, myrcene	MIC ≥ 0.8 (%*v*/*v*)			[93]
Enterococcus *, Staphylococcus *, and *Bacillus* species *	EO	MIC ≥ 0.5 µg/mL	Ampicillin, Ciprofloxacin	MIC ≥ 0.25 µg/mL	[94]
Listeria *monocytogenes* strains *	EO	MIC/MBC 2.5–5.0 μL/mL			[110]
L. monocytogenes *	EO	MIC ≥ 1 µg/mL	Ampicillin	MIC ≥ 0.25 µg/mL	[94]
L. monocytogenes *	EO, α-pinene, Myrcene	MBC ≥ 1024 µg/mL			[111]
Lancefield Group A *Streptococcus* sp.	Leaf extract	MIC 20 mg/mL MBC 30 mg/mL			[113]
MRSA biofilms *	Seed extract	MIC 1 mg/mL			[101]
MSSA	CBCA	MIC 7.8 µM			[52]
MSSA, VISE, *Staphylococcus epidermidis*, *Staphylococcus pyogenes*, *Enterococcus faecalis*, *Cutibacterium acnes, Clostridioides difficile*	CBD	MIC 0.5–4.0 µg/mL	Vancomycin, Daptomycin, Trimethoprim, Mupirocin, Clindamycin, Levofloxacin, Meropenem, Gentamicin, Erythromycin, Tetracycline, Mupirocin	MIC 0.03–64.0 µg/mL	[49]
*Mycobacterium intracellulare*	CBG	IC_50_ 15 µg/mL			[114]
*S. aureus*	4-acetoxy-2-geranyl-5-hydroxy-3-n-pentylphenol, 8-hydroxycannabinolic acid A	IC_50_ 3.5 µM	Ciprofloxacin	IC_50_ 0.4 µM	[96]
*S. aureus*	Aqueous extract	MIC 3.57 mg/mL	Ciprofloxacin	MIC 0.62 µg/mL	[99]
*S. aureus*	Methanol extract	MIC 25 µg/mL			[67]
*S. aureus* (including multi drug resistant *S. aureus* 104)	EO	MIC 8 mg/mL			[61]
*S. aureus* (mature and pre-formed biofilms)	EO	MBEC 24 mg/mL			[61]
*S. aureus* and *E. faecalis*	Seed extract	MIC 1 mg/mL			[101]
*S. aureus* biofilm *	EO	MIC 0.5 mg/mL			[101]
*S. aureus* planktonic cells *	EO	MIC 1 mg/mL			[101]
S. aureus *	EO	MIC 1.25–5.0 µg/mL			[110]
S. aureus *	EO	MIC 1–4 µg/mL	Ciprofloxacin	MIC 0.5–16.0 µg/mL	[94]
*S. aureus*, *S. epidermidis*	CBD, CBDA	MIC 1–4 µg/mL	Torbamycin, Meropenem, Ofloxacin	MIC 0.06–0.5 µg/mL	[50]
SA-1199B (MDR), RN4220 (Macrolide-resistant), XU212 (Tetracycline-resistant)	CBD, CBC, THC, CBG, CBN, Carboxylated versions, Abnormal cannabinoids	MIC 0.5–4.0 µg/mL			[95]
*Staphylococcus* species	THC, CBD	MIC 1–5 µg/mL			[115]
*Staphylococcus*, *Lactococcus* and *Bacillus* species	CBD, CBN, CBC, CBDV and Δ^1 & 9^-THC	IC_50_ 2.6–9.2 µM	Ciprofloxacin	IC_50_ 0.003–2.4 µM	[97]
***Gram-ve***					
*Moraxella catarrhalis*, *Neisseria meningitidis* and *Legionella pneumophila*	CBD	MIC 0.25–1.0 µg/mL	Vancomycin, Levofloxacin, Meropenem, Gentamicin	MIC 0.03–32 µg/mL	[49]
*Pectobacterium carotovorum* subsp. carotovorum *	EO, α-humulene, α-pinene, β-pinene, myrcene	MIC ≥ 1.24 (%*v*/*v*)			[93]
*Pseudomonas fluorescens* and *Xanthobacter flavus*	CBD, CBN, CBC, CBDV and Δ^1 & 9^-THC	IC_50_ 3.1–9.3 µM	Ciprofloxacin	IC_50_ 0.15–2.3 µM	[97]
*Pseudomonas* species	EO(s) and Terpenes	MIC 1.05–1.97 (%*v*/*v)*			[93]

**Table 3:** Activity of cannabinoids and *C. sativa* against fungi.

Pathogen	Compound/Extract/EO	Activity	Reference Antibiotic		Ref
			Antibiotic	Activity	
*Candida albicans*	Extract	MIC 0.25 mg/mL			[124]
*C. albicans*	Extract	MIC 1.42 mg/mL	Fluconazole	MIC 2 mg/mL	[99]
*C. albicans*	4-terpenyl cannabinolate	MIC 8.5 µg/mL			[125]
*C. albicans*	8-hydroxycannabinol	IC_50_ 4.6 µM	Amphotericin B	IC_50_ 0.3 µM	[96]
*C. albicans*	Cannabis and ginger blend	MIC 4.69 mg/mL			[126]
*C. albicans*	CBDV	IC_50_ 11.9 mM	Nystatin	IC_50_ 1.50 mM	[97]
*C. albicans*	CBNA	IC_50_ 8.5 µg/mL			[125]
*Candida krusei*	Cannabinoids	IC_50_ 53.4–60.5 µM	amphotericin B	IC_50_ 0.7 µM	[96]
*Candida neoformans*	β-caryophyllene/oxide	IC_50_ 1.18–19.4 µg/mL			[98]
*Candida* species	β-caryophyllene	MIC 1.45–10.0 µg/mL			[98]
*Plasmodium falciparum*	Cannabinoids	IC_50_ 4.0–6.7 µM	Chloroquine	IC_50_ 0.1–0.5 µM	[96]
*P. falciparum*	CBNA	IC_50_ 2.4–2.7 µg/mL			[125]
*Trichophyton* and *Arthroderma* species	EO	MIC 0.312–6.3 µg/mL	Griseofulvin	MIC 1.26 to >8.0 µg/mL	[123]

**Table 4:** Efficacy of cannabinoids against virus proliferation.

Virus	Compound	Activity (µM)	Ref
Epstein–Barr virus (EBV)	THC	IC_50_ 3.0	[140]
Hepatitis C virus (HCV)	CBD	EC_50_ 3.16	[143]
Herpes simplex virus (HSV)	THC	IC_50_ 1.9	[140]
Kaposi sarcoma associated herpesvirus (KSHV)	THC	IC_50_ 3.3	[140]
KSHV	CBD	IC_50_ 2.08	[144]
Murine gamma herpesvirus 68 (MHV)	THC	IC_50_ 1.9	[140]

**Table 5:** Antiviral activity of cannabinoids against Corona Virus.

Corona Virus Group	Compound	Activity (µM)	Reference Drug	Activity (μM)	Ref
hCov-OC43	Caflanone	EC_50_ 0.42			[184]
SARS-CoV-2 (spike positive)	CBD	EC_50_ 0.64–1.79			[195]
SARS-CoV-2 in A549-ACE2	7-OH-CBD	EC_50_ 3.6			[195]
SARS-CoV-2	CBD, THC, CBN, CBDA, THCA	IC_50_ 7.91–37.61	Remdesivir, Lopinavir and Chloroquine	IC_50_ 8.17–13.16	[189]

## Data Availability

Data available upon request.

## Abbreviations

ACE2	Angiotensin-converting enzyme 2
AIDS	Acquired immunodeficiency syndrome
ARDS	Acute respiratory distress syndrome
CBC	Cannabichromene
CBCA	Cannabichromenic acid
CBD	Cannabidiol
CBDA	Cannabidiolic acid
CBDV	Cannabidivarin
CBDVM	Cannabidivarin methyl ester
CBE	Cannabielsoin
CBG	Cannabigerol
CBL	Cannabicyclol
CBN	Cannabinol
CBT	Cannabitriol
CBV	Cannabivarin
CDC	Centers for disease control and prevention
CNS	Central nervous system
DNA	Deoxyribonucleic acid
EC	Endocannabinoid
EO	Essential oil
FDA	Food and drug administration
GNB	Gram-negative bacteria
GPB	Gram-positive bacteria
HMVEC	Human microvascular endothelial cells
HOMO	Highest occupied molecular orbital
IL	Interleukin
KSHV	Kaposi sarcoma associated herpesvirus
LPS	Lipopolysaccharide
LUMO	Lowest unoccupied molecular orbital
MBEC	Minimum biofilm eradication concentration
MD	Molecular dynamic
MIC	Minimum inhibitory concentration
MRSA	Methicillin-resistant *Staphylococcus aureus*
MSSA	Methicillin-susceptible *Staphylococcus aureus*
MV	Membrane vesicle
PBP	Penicillin-binding proteins
QIDP	Qualified infectious disease product
QSAR	Quantitative structure-activity relationship
RNA	Ribonucleic acid
R&D	Research and Development
SARS-CoV	Severe acute respiratory syndrome coronavirus
THC	Tetrahydrocannabinol
THCA	Tetrahydrocannabinolic acid
TMPRSS2	Transmembrane protease, serine 2
TNF-α	Tumor necrosis factor
VISA	Vancomycin-intermediate *Staphylococcus aureus*
VRSA	Vancomycin-resistant *Staphylococcus aureus*
WHO	World health organization

## References

[1] Burnett-Boothroyd S., McCarthy B. (2011). Antimicrobial treatments of textiles for hygiene and infection control applications: An industrial perspective. Textiles for Hygiene and Infection Control.

[2] Shahid M., Sobia F., Sahai A., Tripathi T., Singh A., Shahzad A., Khan H.M. (2009). Umesh Plant Natural Products as a Potential Source for Antibacterial Agents: Recent Trends. Anti-Infect. Agents Med. Chem..

[3] Ashkenazi S. (2012). Beginning and possibly the end of the antibiotic era. J. Paediatr. Child Health.

[4] Hutchings M.I., Truman A., Wilkinson B. (2019). Editorial overview: Antimicrobials: Tackling AMR in the 21st century. Curr. Opin. Microbiol..

[5] Prestinaci F., Pezzotti P., Pantosti A. (2015). Antimicrobial resistance: A global multifaceted phenomenon. Pathog. Glob. Health.

[6] Abraham E.P., Chain E. (1940). An Enzyme from Bacteria able to Destry penicillin. Nature.

[7] Ventola C.L. (2015). The Antibiotic Resistance Crisis Part 1: Causes and Threats. Pharm. Ther..

[8] Antimicrobial Resistance. https://www.who.int/news-room/fact-sheets/detail/antimicrobial-resistance.

[9] CDC 2019 AR Threats Report. https://www.cdc.gov/drugresistance/biggest_threats.html.

[10] O’Neill J. Review on Antimicrobial Resistance. https://amr-review.org/background.html.

[11] Ledingham K., Hinchliffe S., Jackson M., Thomas F., Tomson G. (2019). Antibiotic resistance: Using a cultural contexts of health approach to address a global health challenge. WHO Reg. Off. Eur..

[12] Aminov R.I. (2010). A Brief History of the Antibiotic Era: Lessons Learned and Challenges for the Future. Front. Microbiol..

[13] Wright G.D. (2014). Something old, something new: Revisiting natural products in antibiotic drug discovery. Can. J. Microbiol..

[14] Piddock L. (2012). The crisis of no new antibiotics—What is the way forward?. Lancet Infect. Dis..

[15] WHO (2017). Global Priority List of Antibiotic-Resistant Bacteria to Guide Research, Discovery, and Development of New Antibiotics.

[16] John Hopkins University. https://coronavirus.jhu.edu/.

[17] Zuardi A.W. (2006). History of cannabis as a medicine: A review. Rev. Bras. Psiquiatr..

[18] Kabelik J., Krejci Z., Santavy F. (1960). Hemp as a medicament. Bull. Narc..

[19] Pisanti S., Malfitano A.M., Ciaglia E., Lamberti A., Ranieri R., Cuomo G., Abate M., Faggiana G., Proto M.C., Fiore D. (2017). Cannabidiol: State of the art and new challenges for therapeutic applications. Pharmacol. Ther..

[20] Gonçalves J., Rosado T., Soares S., Simão A.Y., Caramelo D., Luís Â., Fernández N., Barroso M., Gallardo E., Duarte A.P. (2019). Cannabis and Its Secondary Metabolites: Their Use as Therapeutic Drugs, Toxicological Aspects, and Analytical Determination. Medicines.

[21] Nagarkatti P., Pandey R., Rieder S.A., Hegde V.L., Nagarkatti M. (2009). Cannabinoids as novel anti-inflammatory drugs. Future Med. Chem..

[22] Downer E.J. (2020). Anti-inflammatory Potential of Terpenes Present in *Cannabis sativa* L.. ACS Chem. Neurosci..

[23] Gallily R., Yekhtin Z., Hanuš L.O. (2018). The Anti-Inflammatory Properties of Terpenoids from Cannabis. Cannabis Cannabinoid Res..

[24] Bonini S.A., Premoli M., Tambaro S., Kumar A., Maccarinelli G., Memo M., Mastinu A. (2018). Cannabis sativa: A comprehensive ethnopharmacological review of a medicinal plant with a long history. J. Ethnopharmacol..

[25] Paland N., Pechkovsky A., Aswad M., Hamza H., Popov T., Shahar E., Louria-Hayon I. (2021). The Immunopathology of COVID-19 and the Cannabis Paradigm. Front. Immunol..

[26] Hanuš L.O., Hod Y. (2020). Terpenes/Terpenoids in Cannabis: Are They Important?. Med. Cannabis Cannabinoids.

[27] Tahamtan A., Tavakoli-Yaraki M., Rygiel T.P., Mokhtari-Azad T., Salimi V. (2016). Effects of Cannabinoids and their Receptors on Viral Infections. J. Med. Virol..

[28] Beji C., Loucif H., Telittchenko R., Olagnier D., Dagenais-Lussier X., Van Grevenynghe J. (2020). Cannabinoid-Induced Immunomodulation during Viral Infections: A Focus on Mitochondria. Viruses.

[29] Reiss C.S. (2010). Cannabinoids and Viral Infections. Pharmaceuticals.

[30] Klein T.W., Friedman H., Specter S. (1998). Marijuana, immunity and infection. J. Neuroimmunol..

[31] Cabral G.A., Russo E.B. (2002). Marijuana and Cannabinoids: Effects on infections, immunity, and AIDS. Cannabis Therapeutics in HIV/AIDS.

[32] Tagne A.M., Pacchetti B., Sodergren M., Cosentino M., Marino F. (2020). Cannabidiol for Viral Diseases: Hype or Hope?. Cannabis Cannabinoid Res..

[33] Chianese G., Taglialatela-Scafati O. (2011). Cannabinoids: Occurrence and Medicinal Chemistry. Curr. Med. Chem..

[34] Nascimento G.G.F., Locatelli J., Freitas P.C., Silva G.L. (2000). Antibacterial activity of plant extracts and phytochemicals on antibiotic-resistant bacteria. Braz. J. Microbiol..

[35] Marcu J.P. (2016). An Overview of Major and Minor Phytocannabinoids.

[36] Krejci Z. (1950). Hemp as a Medicament. Ph.D. Thesis.

[37] Krejci Z. (1952). Antibacterial action of Canabis indica. Lek. List..

[38] Krejci Z. (1959). Hanf (Cannabis sativa) -Antibiotisches Heilmittel. 2. Mitteilung: Methodik und Ergebnisse der bakteriologischen Untersuchungen und vorläufige klinische Erfahrungen. Pharmazie.

[39] Ferenczy L. (1956). Antbacterial substances in seeds. Nature.

[40] Ferenczy L., Gracza L., Jakobey I. (1958). An antibacterial preparatum from hemp (*Cannabis sativa* L.). Naturwissenschaften.

[41] Boucher H.W., Talbot G.H., Bradley J.S., Edwards J.E., Gilbert D., Rice L.B., Scheld M., Spellberg B., Bartlett J. (2009). Bad Bugs, No Drugs: No ESKAPE! An Update from the Infectious Diseases Society of America. Clin. Infect. Dis..

[42] Nannini E., Murray B.E., Arias C.A. (2010). Resistance or decreased susceptibility to glycopeptides, daptomycin, and linezolid in methicillin-resistant Staphylococcus aureus. Curr. Opin. Pharmacol..

[43] Farha M.A., El-Halfawy O.M., Gale R.T., MacNair C.R., Carfrae L.A., Zhang X., Jentsch N.G., Magolan J., Brown E.D. (2020). Uncovering the Hidden Antibiotic Potential of Cannabis. ACS Infect. Dis..

[44] Lewis K. (2006). Persister cells, dormancy and infectious disease. Nat. Rev. Genet..

[45] Otto M. (2013). Staphylococcal Infections: Mechanisms of Biofilm Maturation and Detachment as Critical Determinants of Pathogenicity. Annu. Rev. Med..

[46] Mah T.-F.C., O’Toole G.A. (2001). Mechanisms of biofilm resistance to antimicrobial agents. Trends Microbiol..

[47] Conlon B.P., Rowe S.E., Gandt A.B., Nuxoll A.S., Donegan N.P., Zalis E.A., Clair G., Adkins J.N., Cheung A.L., Lewis K. (2016). Persister formation in Staphylococcus aureus is associated with ATP depletion. Nat. Microbiol..

[48] Severin A., Tabei K., Tenover F., Chung M., Clarke N., Tomasz A. (2004). High Level Oxacillin and Vancomycin Resistance and Altered Cell Wall Composition in Staphylococcus aureus Carrying the Staphylococcal mecA and the Enterococcal vanA Gene Complex. J. Biol. Chem..

[49] Blaskovich M.A.T., Kavanagh A.M., Elliott A.G., Zhang B., Ramu S., Amado M., Lowe G.J., Hinton A.O., Pham D.M.T., Zuegg J. (2021). The antimicrobial potential of cannabidiol. Commun. Biol..

[50] Martinenghi L.D., Jønsson R., Lund T., Jenssen H. (2020). Isolation, Purification, and Antimicrobial Characterization of Cannabidiolic Acid and Cannabidiol from *Cannabis sativa* L.. Biomolecules.

[51] Wassmann C.S., Højrup P., Klitgaard J.K. (2020). Cannabidiol is an effective helper compound in combination with bacitracin to kill Gram-positive bacteria. Sci. Rep..

[52] Galletta M., Reekie T., Nagalingam G., Bottomley A., Harry E., Kassiou M., Triccas J. (2020). Rapid Antibacterial Activity of Cannabichromenic Acid against Methicillin-Resistant *Staphylococcus aureus*. Antibiotics.

[53] Mascio C.T.M., Alder J.D., Silverman J.A. (2007). Bactericidal Action of Daptomycin against Stationary-Phase and Nondividing Staphylococcus aureus Cells. Antimicrob. Agents Chemother..

[54] Russo E.B. (2011). Taming THC: Potential cannabis synergy and phytocannabinoid-terpenoid entourage effects. Br. J. Pharmacol..

[55] Chakraborty S., Afaq N., Singh N., Majumdar S. (2018). Antimicrobial activity of Cannabis sativa, Thuja orientalis and Psidium guajava leaf extracts against methicillin-resistant Staphylococcus aureus. J. Integr. Med..

[56] Nashra A., Sujatha R., Sameer D., Bagoliwal A., Mishra V., Kumar A., Majid A. (2018). Comparative Evaluation of Antibacterial Efficacy of Cannabis Sativa, Allium Sativum, Allium Cepa, Thuja Orientalis and Psidium Guajava against Drug Resistance Pathogens. Int. J. Health Sci. Res..

[57] Torres J.A., Villegas M.V., Quinn J.P. (2007). Current concepts in antibiotic-resistant Gram-negative bacteria. Expert Rev. Anti-Infect. Ther..

[58] Exner M., Bhattacharya S., Christiansen B., Gebel J., Goroncy-Bermes P., Hartemann P., Heeg P., Ilschner C., Kramer A., Larson E. (2017). Antibiotic resistance: What is so special about multidrug-resistant Gram-negative bacteria? Antibiotikaresistenz: Was ist so besonders an den Gram-negativen. GMS Hyg. Infect. Control.

[59] Miller S.I. (2016). Antibiotic Resistance and Regulation of the Gram-Negative Bacterial Outer Membrane Barrier by Host Innate Immune Molecules. mBio.

[60] Nafis A., Kasrati A., Jamali C.A., Mezrioui N., Setzer W., Abbad A., Hassani L. (2019). Antioxidant activity and evidence for synergism of *Cannabis sativa* (L.) essential oil with antimicrobial standards. Ind. Crops Prod..

[61] Zengin G., Menghini L., Di Sotto A., Mancinelli R., Sisto F., Carradori S., Cesa S., Fraschetti C., Filippi A., Angiolella L. (2018). Chromatographic Analyses, In Vitro Biological Activities, and Cytotoxicity of *Cannabis sativa* L. Essential Oil: A Multidisciplinary Study. Molecules.

[62] Shah S.B., Sartaj L., Hussain S., Ullah N., Idrees M., Shaheen A., Javed M.S., Aslam M.K. (2019). In-vitro evaluation of antimicrobial, antioxidant, alpha-amylase inhibition and cytotoxicity properties of Cannabis sativa. Adv. Tradit. Med..

[63] Kosgodage U.S., Matewele P., Awamaria B., Kraev I., Warde P., Mastroianni G., Nunn A.V., Guy G.W., Bell J.D., Inal J. (2019). Cannabidiol Is a Novel Modulator of Bacterial Membrane Vesicles. Front. Cell. Infect. Microbiol..

[64] Delcour A.H. (2009). Outer membrane permeability and antibiotic resistance. Biochim. et Biophys. Acta (BBA)—Proteins Proteom..

[65] Nagakubo T., Nomura N., Toyofuku M. (2019). Cracking Open Bacterial Membrane Vesicles. Front. Microbiol..

[66] Pang Z., Raudonis R., Glick B.R., Lin T.-J., Cheng Z. (2018). Antibiotic resistance in Pseudomonas aeruginosa: Mechanisms and alternative therapeutic strategies. Biotechnol. Adv..

[67] Ali E.M.M., Almagboul A.Z.I., Khogali S.M.E., Gergeir U.M.A. (2012). Antimicrobial Activity of *Cannabis sativa* L.. Chin. Med..

[68] Anjum M., Arooj Z.-E.-, Azam S., Rehman P., Khadim J., Anjum M. (2018). Evaluation of antimicrobial activity and ethnobotanical study of *Cannabis sativa* L.. Pure Appl. Biol..

[69] Kourmouli A., Valenti M., Van Rijn E., Beaumont H.J.E., Kalantzi O.-I., Schmidt-Ott A., Biskos G. (2018). Can disc diffusion susceptibility tests assess the antimicrobial activity of engineered nanoparticles?. J. Nanoparticle Res..

[70] Klančnik A., Piskernik S., Jeršek B., Možina S.S. (2010). Evaluation of diffusion and dilution methods to determine the antibacterial activity of plant extracts. J. Microbiol. Methods.

[71] Mathur P., Singh A., Srivastava V.R., Singh D., Mishra Y. (2013). Antimicrobial activity of indigenous wildly growing plants: Potential source of green antibiotics. Afr. J. Microbiol. Res..

[72] Naveed M., Khan T.A., Ali I., Hassan A., Ali H., Ud Z., Hassan Z., Tabassum S., Majid A., Rehman M.U. (2014). In vitro antibacterial activity of *Cannabis sativa* leaf extracts to some selective pathogenicbacterial strains. Int. J. Biosci. (IJB).

[73] Lone T.A., Lone R.A. (2012). Extraction of cannabinoids from *Cannabis sativa* L plant and its potential antimicrobial activity. Univers. J. Med. Dent..

[74] Mkpenie V.N., Essien E.E., Udoh I.I. (2012). Effect of extraction conditions on total polyphenol contents, antioxidant and antimicrobial activities of *Cannabis sativa* L.. Electron. J. Environ. Agric. Food Chem..

[75] Elsohly H.N., Turner C.E., Clark A.M., Elsohly M.A. (1982). Synthesis and Antimicrobial Activities of Certain Cannabichromene and Cannabigerol Related Compounds. J. Pharm. Sci..

[76] Ullah S., Jan G., Gul F., Khan S., Husna H., Sher J., Abidullah S. (2018). Phytochemistry and antibacterial activities of some selected plants of war affected area of bajaur agency, pakistan. J. Pharmacogn. Phytochem..

[77] Nadir I., Rana N.F., Ahmad N.M., Tanweer T., Batool A., Taimoor Z., Riaz S., Ali S.M. (2020). Cannabinoids and Terpenes as an Antibacterial and Antibiofouling Promotor for PES Water Filtration Membranes. Molecules.

[78] Kim S.-Y., Kang D.-H., Kim J.-K., Ha Y.-G., Hwang J.Y., Kim T., Lee S.-H. (2010). Antimicrobial Activity of Plant Extracts Against *Salmonella typhimurium*, Escherichia coli O157:H7, and Listeria monocytogenes on Fresh Lettuce. J. Food Sci..

[79] Novak J., Zitterl-Eglseer K., Deans S.G., Franz C.M. (2001). Essential oils of different cultivars of *Cannabis sativa* L. and their antimicrobial activity. Flavour Fragr. J..

[80] Nasrullah S., Rahman K., Ikram M., Nisar M., Khan I. (2012). Screening of antibacterial activity of medicinal plants. Int. J. Pharm. Sci. Rev. Res..

[81] Sharma C., Kaur S., Chaudhry S., Aman R. (2015). Antimicrobial Potential of Three Common Weeds of Kurukshetra: An in vitro Study. Res. J. Microbiol..

[82] Ali M., Romman M., Parvez R., Shuaib M., Bahadur S., Khalil A.A.K., Khan M., Haq F., Jan S., Hayat S.S.S. (2020). Anti-bacterial activity of *Cannabis sativa* Linn. leaf extracts against different pathogenic bacterial strains. Biosci. Res..

[83] Zheljazkov V.D., Sikora V., Dincheva I., Kačániová M., Astatkie T., Semerdjieva I.B., Latkovic D. (2020). Industrial, CBD, and Wild Hemp: How Different Are Their Essential Oil Profile and Antimicrobial Activity?. Molecules.

[84] Mikulcová V., Kašpárková V., Humpolíček P., Buňková L. (2017). Formulation, Characterization and Properties of Hemp Seed Oil and Its Emulsions. Molecules.

[85] Oyedemi B.M. (2014). Antiplasmid and Antimicrobial Activities of Synthetic and Natural Products from Selected Medicinal Plants.

[86] Raleigh E., Low K. (2013). Conjugation. Brenner’s Encycl. Genet. Second Ed..

[87] Spengler G., Molnar A., Schelz Z., Amaral L., Sharples D., Molnar J. (2006). The Mechanism of Plasmid Curing in Bacteria. Curr. Drug Targets.

[88] Molnár J., Csiszár K., Nishioka I., Shoyama Y. (1986). The effects of cannabispiro compounds and tetrahydrocannabidiolic acid on the plasmid transfer and maintenance in *Escherichia coli*. Acta Microbiol. Hung..

[89] Feldman M., Smoum R., Mechoulam R., Steinberg D. (2018). Antimicrobial potential of endocannabinoid and endocannabinoid-like compounds against methicillin-resistant Staphylococcus aureus. Sci. Rep..

[90] Feldman M., Smoum R., Mechoulam R., Steinberg D. (2020). Potential combinations of endocannabinoid/endocannabinoid-like compounds and antibiotics against methicillin-resistant Staphylococcus aureus. PLoS ONE.

[91] Veringa E.M., Ferguson D.A., Lambe D.W., Verhoef J. (1989). The role of glycocalyx in surface phagocytosis of *Baeteroides* spp., in the presence and absence of clindamycin. J. Antimicrob. Chemother..

[92] Nazir R., Rehman S., Nisa M., Baba U. (2019). ali Exploring bacterial diversity: From cell to sequence. Freshwater Microbiology: Perspectives of Bacterial Dynamics in Lake Ecosystems.

[93] Nissen L., Zatta A., Stefanini I., Grandi S., Sgorbati B., Biavati B., Monti A. (2010). Characterization and antimicrobial activity of essential oils of industrial hemp varieties (*Cannabis sativa* L.). Fitoterapia.

[94] Iseppi R., Brighenti V., Licata M., Lambertini A., Sabia C., Messi P., Pellati F., Benvenuti S. (2019). Chemical Characterization and Evaluation of the Antibacterial Activity of Essential Oils from Fibre-Type *Cannabis sativa* L. (Hemp). Molecules.

[95] Appendino G., Gibbons S., Giana A., Pagani A., Grassi G., Stavri M., Smith E., Rahman M. (2008). Antibacterial Cannabinoids from Cannabis sativa: A Structure−Activity Study. J. Nat. Prod..

[96] Radwan M.M., ElSohly M.A., Slade D., Ahmed S.A., Khan I.A., Ross S.A. (2009). Biologically Active Cannabinoids from High-Potency Cannabis sativa. J. Nat. Prod..

[97] Nalli Y., Arora P., Riyaz-Ul-Hassan S., Ali A. (2018). Chemical investigation of *Cannabis sativa* leading to the discovery of a prenylspirodinone with anti-microbial potential. Tetrahedron Lett..

[98] Wanas A.S., Radwan M.M., Mehmedic Z., Jacob M., Khan I.A., Elsohly M.A. (2015). Antifungal activity of the volatiles of high potency *Cannabis sativa* L. Against Cryptococcus neoformans. Rec. Nat. Prod..

[99] Ferrante C., Recinella L., Ronci M., Menghini L., Brunetti L., Chiavaroli A., Leone S., Di Iorio L., Carradori S., Tirillini B. (2019). Multiple pharmacognostic characterization on hemp commercial cultivars: Focus on inflorescence water extract activity. Food Chem. Toxicol..

[100] Elhendawy M.A., Wanas A.S., Radwan M.M., Azzaz N.A., Toson E.S., ElSohly M.A. (2018). Chemical and Biological Studies of *Cannabis sativa* Roots. Med. Cannabis Cannabinoids.

[101] Frassinetti S., Gabriele M., Moccia E., Longo V., Di Gioia D. (2020). Antimicrobial and antibiofilm activity of *Cannabis sativa* L. seeds extract against Staphylococcus aureus and growth effects on probiotic *Lactobacillus* spp.. LWT.

[102] Nocera F.P., Mancini S., Najar B., Bertelloni F., Pistelli L., De Filippis A., Fiorito F., De Martino L., Fratini F. (2020). Antimicrobial Activity of Some Essential Oils against Methicillin-Susceptible and Methicillin-Resistant *Staphylococcus pseudintermedius*-Associated Pyoderma in Dogs. Animals.

[103] Radošević A., Kupinić M., Grlić L., Kupini M. (1962). Antibiotic Activity of Various Types of Cannabis Resin. Nature.

[104] Kabelik V.J. (1958). Hanf (Cannabis sativa)—Antibiotisches Heilmittel. 1. Mitteilung: Hanf in der Alt- und Volksmedizin. Pharmazie.

[105] Wasim K., Haq I., Ashraf M. (1995). Antimicrobial studies of the leaf of *Cannabis sativa* L.. Pak. J. Pharm. Sci..

[106] Borchardt J.R., Wyse D.L., Sheaffer C.C., Kauppi K.L., Fulcher R.G., Ehlke N.J., Biesboer D.D., Bey R.F. (2008). Antimicrobial activity of native and naturalized plants of Minnesota and Wisconsin. J. Med. Plants Res..

[107] Zheljazkov V.D., Sikora V., Semerdjieva I.B., Kačániová M., Astatkie T., Dincheva I. (2020). Grinding and Fractionation during Distillation Alter Hemp Essential Oil Profile and Its Antimicrobial Activity. Molecules.

[108] Lelario F., Scrano L., De Franchi S., Bonomo M.G., Salzano G., Milan S., Milella L., Bufo S.A. (2018). Identification and antimicrobial activity of most representative secondary metabolites from different plant species. Chem. Biol. Technol. Agric..

[109] Viswanath H., Bhat K.A., Bhat N., Wani T., Mughal M.N. (2018). Antibacterial Efficacy of Aqueous Plant Extracts against Storage Soft Rot of Potato Caused by Erwinia carotovora. Int. J. Curr. Microbiol. Appl. Sci..

[110] Pellegrini M., Palmieri S., Ricci A., Serio A., Paparella A., Sterzo C.L. (2020). In vitro antioxidant and antimicrobial activity of *Cannabis sativa* L. cv ‘Futura 75’ essential oil. Nat. Prod. Res..

[111] Marini E., Magi G., Ferretti G., Bacchetti T., Giuliani A., Pugnaloni A., Rippo M.R., Facinelli B. (2018). Attenuation of Listeria monocytogenes Virulence by *Cannabis sativa* L. Essential Oil. Front. Cell. Infect. Microbiol..

[112] Turner C.E., Elsohly M.A. (1981). Biological Activity of Cannabichromene, its Homologs and Isomers. J. Clin. Pharmacol..

[113] Anumudu C.K., Akpaka M.N., Anumudu I.C. (2020). Antimicrobial activity of *Cannabis sativa* extracts on Lancefield Group A Streptococcus species associated with streptococcal pharyngitis (strep throat). Afr. J. Biol. Sci..

[114] Radwan M.M., Ross S.A., Slade D., Ahmed S.A., Zulfiqar F., ElSohly M.A. (2008). Isolation and Characterization of New Cannabis Constituents from a High Potency Variety. Planta Med..

[115] Van Klingeren B., Ham M.T. (1976). Antibacterial activity of Δ9-tetrahydrocannabinol and cannabidiol. Antonie van Leeuwenhoek.

[116] Nola I., Kostović K., Oremović L., Soldo-Belić A., Lugović L. (2003). Candida infections today—How big is the problem?. Acta Dermatovenerol. Croat..

[117] Aleksic V., Knezevic P. (2014). Antimicrobial and antioxidative activity of extracts and essential oils of *Myrtus communis* L.. Microbiol. Res..

[118] Dabur R., Singh H., Chhillar A., Ali M., Sharma G. (2004). Antifungal potential of Indian medicinal plants. Fitoterapia.

[119] Kuete V. (2010). Potential of Cameroonian Plants and Derived Products against Microbial Infections: A Review. Planta Med..

[120] Nobile C.J., Johnson A.D. (2015). Candida albicans Biofilms and Human Disease. Annu. Rev. Microbiol..

[121] Tsui C., Kong E.F., Jabra-Rizk M.A. (2016). Pathogenesis ofCandida albicansbiofilm. Pathog. Dis..

[122] Khan I.H., Javaid A. (2020). Antifungal activity of leaf extract of *Cannabis sativa* against Aspergillus flavipes. Pak. J. Weed Sci. Res..

[123] Orlando G., Adorisio S., Delfino D., Chiavaroli A., Brunetti L., Recinella L., Leone S., D’Antonio M., Zengin G., Acquaviva A. (2021). Comparative Investigation of Composition, Antifungal, and Anti-Inflammatory Effects of the Essential Oil from Three Industrial Hemp Varieties from Italian Cultivation. Antibiotics.

[124] Aboul-ela M.A., Bahaa N., Din E. (1995). Antimicrobial Evaluation of Extracts from some Yemeni Plants. Alexander J. Pharm. Sci..

[125] Ahmed S.A., Ross S.A., Slade D., Radwan M.M., Zulfiqar F., ElSohly M.A. (2008). Cannabinoid Ester Constituents from High-Potency Cannabis sativa. J. Nat. Prod..

[126] Žitek T., Leitgeb M., Golle A., Dariš B., Knez Ž., Hrnčič M.K. (2020). The Influence of Hemp Extract in Combination with Ginger on the Metabolic Activity of Metastatic Cells and Microorganisms. Molecules.

[127] Miller A.M., Stella N. (2008). CB2 receptor-mediated migration of immune cells: It can go either way. Br. J. Pharmacol..

[128] Yao B., Mackie K. (2009). Endocannabinoid Receptor Pharmacology. Curr. Top. Behav. Neurosci..

[129] Reddy P.M., Maurya N., Velmurugan B.K. (2019). Medicinal Use of Synthetic Cannabinoids—A Mini Review. Curr. Pharmacol. Rep..

[130] Herrera R.A., Oved J.H., Reiss C.S. (2008). Disruption of IFN-γ–Mediated Antiviral Activity in Neurons: The Role of Cannabinoids. Viral Immunol..

[131] Rice W., Shannon J.M., Burton F., Fiedeldey D. (1997). Expression of a brain-type cannabinoid receptor (CB1) in alveolar Type II cells in the lung: Regulation by hydrocortisone. Eur. J. Pharmacol..

[132] Van Der Poorten D., Shahidi M., Tay E., Sesha J., Tran K., McLeod D., Milliken J.S., Ho V., Hebbard L.W., Douglas M.W. (2010). Hepatitis C Virus Induces the Cannabinoid Receptor 1. PLoS ONE.

[133] Nichols J.M., Kaplan B.L. (2020). Immune Responses Regulated by Cannabidiol. Cannabis Cannabinoid Res..

[134] Walter L., Stella N. (2004). Cannabinoids and neuroinflammation. Br. J. Pharmacol..

[135] D’Addario C., Di Francesco A., Pucci M., Agrò A.F., Maccarrone M. (2013). Epigenetic mechanisms and endocannabinoid signalling. FEBS J..

[136] Rock R.B., Gekker G., Hu S., Sheng W.S., Cabral G.A., Martin B.R., Peterson P.K. (2006). WIN55,212-2-Mediated Inhibition of HIV-1 Expression in Microglial Cells: Involvement of Cannabinoid Receptors. J. Neuroimmune Pharmacol..

[137] Ramirez S.H., Reichenbach N.L., Fan S., Rom S., Merkel S.F., Wang X., Ho W.-Z., Persidsky Y. (2013). Attenuation of HIV-1 replication in macrophages by cannabinoid receptor 2 agonists. J. Leukoc. Biol..

[138] Molina P.E., Winsauer P., Zhang P., Walker E., Birke L., Amedee A., Stouwe C.V., Troxclair D., McGoey R., Varner K. (2011). Cannabinoid Administration Attenuates the Progression of Simian Immunodeficiency Virus. AIDS Res. Hum. Retrovir..

[139] Abubakar Y.U., Taura D.W., Yushau M., Muhammad A.U. (2020). An in ovo investigation on antiviral activity of *Cannabis sativa* extracts against Newcastle Disease Virus (NDV). Adv. Pharm. J..

[140] Medveczky M.M., Sherwood T.A., Klein T.W., Friedman H., Medveczky P.G. (2004). Delta-9 tetrahydrocannabinol (THC) inhibits lytic replication of gamma oncogenic herpesviruses in vitro. BMC Med..

[141] Lancz G., Specter S., Brown H.K. (1991). Suppressive Effect of -9-Tetrahydrocannabinol on Herpes Simplex Virus Infectivity In Vitro. Exp. Biol. Med..

[142] Blevins R.D., Dumic M.P. (1980). The Effect of -9-Tetrahydrocannabinol on Herpes Simplex Virus Replication. J. Gen. Virol..

[143] Toyang N.J., Lowe H.I.C., McLaughlin W. (2017). Potential of cannabidiol for the treatment of viral hepatitis. Pharmacogn. Res..

[144] Maor Y., Yu J., Kuzontkoski P.M., Dezube B.J., Zhang X., Groopman J.E. (2012). Cannabidiol Inhibits Growth and Induces Programmed Cell Death in Kaposi Sarcoma-Associated Herpesvirus-Infected Endothelium. Genes Cancer.

[145] Lowe H., Steele B., Bryant J., Fouad E., Toyang N., Ngwa W. (2021). Antiviral Activity of Jamaican Medicinal Plants and Isolated Bioactive Compounds. Molecules.

[146] FDA FDA and Cannabis: Research and Drug Approval Process. https://www.fda.gov/news-events/public-health-focus/fda-and-cannabis-research-and-drug-approval-process.

[147] Wu Y., Ho W., Huang Y., Jin D.-Y., Li S., Liu S.-L., Liu X., Qiu J., Sang Y., Wang Q. (2020). SARS-CoV-2 is an appropriate name for the new coronavirus. Lancet.

[148] Hojyo S., Uchida M., Tanaka K., Hasebe R., Tanaka Y., Murakami M., Hirano T. (2020). How COVID-19 induces cytokine storm with high mortality. Inflamm. Regen..

[149] Mahmudpour M., Roozbeh J., Keshavarz M., Farrokhi S., Nabipour I. (2020). COVID-19 cytokine storm: The anger of inflammation. Cytokine.

[150] Xu X., Han M., Li T., Sun W., Wang D., Fu B., Zhou Y., Zheng X., Yang Y., Li X. (2020). Effective treatment of severe COVID-19 patients with tocilizumab. Proc. Natl. Acad. Sci. USA.

[151] Del Valle D.M., Kim-Schulze S., Huang H.-H., Beckmann N.D., Nirenberg S., Wang B., Lavin Y., Swartz T.H., Madduri D., Stock A. (2020). An inflammatory cytokine signature predicts COVID-19 severity and survival. Nat. Med..

[152] Huang Q., Wu X., Zheng X., Luo S., Xu S., Weng J. (2020). Targeting inflammation and cytokine storm in COVID-19. Pharmacol. Res..

[153] Song Y., Zhang M., Yin L., Wang K., Zhou Y., Zhou M., Lu Y. (2020). COVID-19 treatment: Close to a cure? A rapid review of pharmacotherapies for the novel coronavirus (SARS-CoV-2). Int. J. Antimicrob. Agents.

[154] Lucaciu O., Aghiorghiesei O., Petrescu N.B., Mirica I.C., Benea H.R.C., Apostu D. (2021). In quest of a new therapeutic approach in COVID-19: The endocannabinoid system. Drug Metab. Rev..

[155] Wang B., Kovalchuk A., Li D., Rodriguez-Juarez R., Ilnytskyy Y., Kovalchuk I., Kovalchuk O. (2020). In search of preventative strategies: Novel high-CBD *Cannabis sativa* extracts modulate ACE2 expression in COVID-19 gateway tissues. Aging.

[156] Zhou P., Yang X.-L., Wang X.-G., Hu B., Zhang L., Zhang W., Si H.-R., Zhu Y., Li B., Huang C.-L. (2020). A pneumonia outbreak associated with a new coronavirus of probable bat origin. Nature.

[157] Mollica V., Rizzo A., Massari F. (2020). The pivotal role of TMPRSS2 in coronavirus disease 2019 and prostate cancer. Futur. Oncol..

[158] Hoffmann M., Kleine-Weber H., Schroeder S., Krüger N., Herrler T., Erichsen S., Schiergens T.S., Herrler G., Wu N.-H., Nitsche A. (2020). SARS-CoV-2 Cell Entry Depends on ACE2 and TMPRSS2 and Is Blocked by a Clinically Proven Protease Inhibitor. Cell.

[159] Li M., Chen L., Zhang J., Xiong C., Li X. (2020). The SARS-CoV-2 receptor ACE2 expression of maternal-fetal interface and fetal organs by single-cell transcriptome study. PLoS ONE.

[160] Malinowska B., Baranowska-kuczko M., Kicman A., Schlicker E. (2021). Opportunities, Challenges and Pitfalls of Using Cannabidiol as an Adjuvant Drug in COVID-19. Int. J. Mol. Sci..

[161] Ragia G., Manolopoulos V.G. (2020). Inhibition of SARS-CoV-2 entry through the ACE2/TMPRSS2 pathway: A promising approach for uncovering early COVID-19 drug therapies. Eur. J. Clin. Pharmacol..

[162] Jin Z., Du X., Xu Y., Deng Y., Liu M., Zhao Y., Zhang B., Li X., Zhang L., Peng C. (2020). Structure of Mpro from SARS-CoV-2 and discovery of its inhibitors. Nature.

[163] Zhang L., Lin D., Sun X., Curth U., Drosten C., Sauerhering L., Becker S., Rox K., Hilgenfeld R. (2020). Crystal structure of SARS-CoV-2 main protease provides a basis for design of improved α-ketoamide inhibitors. Science.

[164] COVID-19 Treatment Guidelines Panel Coronavirus Disease 2019 (COVID-19) Treatment Guidelines. https://www.covid19treatmentguidelines.nih.gov.

[165] Rossi F., Tortora C., Argenziano M., Di Paola A., Punzo F. (2020). Cannabinoid Receptor Type 2: A Possible Target in SARS-CoV-2 (CoV-19) Infection?. Int. J. Mol. Sci..

[166] Hill K.P. (2020). Cannabinoids and the Coronavirus. Cannabis Cannabinoid Res..

[167] Onaivi E.S., Sharma V. (2020). Cannabis for COVID-19: Can cannabinoids quell the cytokine storm?. Future Sci. OA.

[168] Esposito G., Pesce M., Seguella L., Sanseverino W., Lu J., Corpetti C., Sarnelli G. (2020). The potential of cannabidiol in the COVID-19 pandemic. Br. J. Pharmacol..

[169] Mamber S.W., Krakowka S., Osborn J., Saberski L., Rhodes R.G., Dahlberg A.E., Pond-Tor S., Fitzgerald K., Wright N., Beseme S. (2020). Can Unconventional Immunomodulatory Agents Help Alleviate COVID-19 Symptoms and Severity?. mSphere.

[170] Costiniuk C.T., Jenabian M.-A. (2020). Acute inflammation and pathogenesis of SARS-CoV-2 infection: Cannabidiol as a potential anti-inflammatory treatment?. Cytokine Growth Factor Rev..

[171] Byrareddy S.N., Mohan M. (2020). SARS-CoV2 induced respiratory distress: Can cannabinoids be added to anti-viral therapies to reduce lung inflammation?. Brain Behav. Immun..

[172] Zhu W., Newton C., Daaka Y., Friedman H., Klein T.W. (1994). delta 9-Tetrahydrocannabinol enhances the secretion of interleukin 1 from endotoxin-stimulated macrophages. J. Pharmacol. Exp. Ther..

[173] Srivastava M.D., Srivastava B., Brouhard B. (1998). Δ9 Tetrahydrocannabinol and cannabidiol alter cytokine production by human immune cells. Immunopharmacology.

[174] Kishimoto S., Kobayashi Y., Oka S., Gokoh M., Waku K., Sugiura T. (2004). 2-Arachidonoylglycerol, an endogenous cannabinoid receptor ligand, induces accelerated production of chemokines in HL-60 cells. J. Biochem..

[175] Anil S.M., Shalev N., Vinayaka A.C., Nadarajan S., Namdar D., Belausov E., Shoval I., Mani K.A., Mechrez G., Koltai H. (2021). Cannabis compounds exhibit anti-inflammatory activity in vitro in COVID-19-related inflammation in lung epithelial cells and pro-inflammatory activity in macrophages. Sci. Rep..

[176] Mormina M., Thakur S., Molleman A., Whelan C.J., Baydoun A. (2006). Cannabinoid signalling in TNF-α induced IL-8 release. Eur. J. Pharmacol..

[177] Abani O., Abbas A., Abbas F., Abbas M., Abbasi S., Abbass H., Abbott A., Abdallah N., Abdelaziz A., Abdelfattah M. (2021). Tocilizumab in patients admitted to hospital with COVID-19 (RECOVERY): Preliminary results of a randomised, controlled, open-label, platform trial. Lancet.

[178] Gremese E., Cingolani A., Bosello S.L., Alivernini S., Tolusso B., Perniola S., Landi F., Pompili M., Murri R., Santoliquido A. (2020). Sarilumab use in severe SARS-CoV-2 pneumonia. EClinicalMedicine.

[179] Gritti G., Raimondi F., Ripamonti D., Riva I., Landi F., Alborghetti L., Frigeni M., Damiani M., Micò C., Fagiuoli S. (2018). IL-6 signalling pathway inactivation with siltuximab in patients with COVID-19 respiratory failure: An observational cohort study. medRxiv.

[180] FDA ok for Phase III Trial of Siltuximab for COVID-19. https://www.thepharmaletter.com/article/fda-ok-for-phase-iii-trial-of-siltuximab-for-covid-19.

[181] Khodadadi H., Salles L., Jarrahi A., Chibane F., Costigliola V., Yu J.C., Vaibhav K., Hess D.C., Dhandapani K.M., Baban B. (2020). Cannabidiol Modulates Cytokine Storm in Acute Respiratory Distress Syndrome Induced by Simulated Viral Infection Using Synthetic RNA. Cannabis Cannabinoid Res..

[182] Salles L., Khodadadi H., Jarrahi A., Ahluwalia M., Paffaro V.A., Costigliola V., Yu J.C., Hess D.C., Dhandapani K.M., Baban B. (2020). Cannabidiol (CBD) modulation of apelin in acute respiratory distress syndrome. J. Cell. Mol. Med..

[183] Lowe H., Steele B., Bryant J., Toyang N., Ngwa W. (2021). Non-Cannabinoid Metabolites of *Cannabis sativa* L. with Therapeutic Potential. Plants.

[184] Ngwa W., Kumar R., Thompson D., Lyerly W., Moore R., Reid T.-E., Lowe H., Toyang N. (2020). Potential of Flavonoid-Inspired Phytomedicines against COVID-19. Molecules.

[185] Mohammed A., Alghetaa H.K., Zhou J., Chatterjee S., Nagarkatti P., Nagarkatti M. (2020). Protective effects of Δ9-tetrahydrocannabinol against enterotoxin-induced acute respiratory distress syndrome are mediated by modulation of microbiota. Br. J. Pharmacol..

[186] Zgair A., Lee J.B., Wong J.C.M., Taha D., Aram J., Di Virgilio D., McArthur J.W., Cheng Y.-K., Hennig I.M., Barrett D.A. (2017). Oral administration of cannabis with lipids leads to high levels of cannabinoids in the intestinal lymphatic system and prominent immunomodulation. Sci. Rep..

[187] Chatow L., Nudel A., Nesher I., Hemo D.H., Rozenberg P., Voropaev H., Winkler I., Levy R., Kerem Z., Yaniv Z. (2021). In Vitro Evaluation of the Activity of Terpenes and Cannabidiol against Human Coronavirus E229. Life.

[188] Crossney J. A Storm of Research Activity into Cannabis and Coronavirus a Closer Look at Expanding Research of Cannabinoids and Terpenes in COVID-19. https://www.cannabissciencetech.com/view/a-storm-of-research-activity-into-cannabis-and-coronavirus-a-closer-look-at-expanding-research-of-cannabinoids-and-terpenes-in-COVID-19.

[189] Raj V., Park J.G., Cho K.-H., Choi P., Kim T., Ham J., Lee J. (2020). Assessment of antiviral potencies of cannabinoids against SARS-CoV-2 using computational and in vitro approaches. Int. J. Biol. Macromol..

[190] Mishra D., Maurya R.R., Kumar K., Munjal N.S., Bahadur V., Sharma S., Singh P., Bahadur I. (2021). Structurally modified compounds of hydroxychloroquine, remdesivir and tetrahydrocannabinol against main protease of SARS-CoV-2, a possible hope for COVID-19: Docking and molecular dynamics simulation studies. J. Mol. Liq..

[191] Sarkar I., Sen G., Bhattyachariya M., Bhattacharyya S., Sen A. (2021). In silico inquest reveals the efficacy of Cannabis in the treatment of post-Covid-19 related neurodegeneration. J. Biomol. Struct. Dyn..

[192] Kielian M. (2020). Enhancing host cell infection by SARS-CoV-2. Science.

[193] Orio L.P., Boschin G., Recca T., Morelli C.F., Ragona L., Francescato P., Arnoldi A., Speranza G. (2017). New ACE-Inhibitory Peptides from Hemp Seed (*Cannabis sativa* L.) Proteins. J. Agric. Food Chem..

[194] Tadayon N., Ramazani A. (2021). A review on the syntheses of Dronabinol and Epidiolex as classical cannabinoids with various biological activities including those against SARS-COV2. J. Iran. Chem. Soc..

[195] Nguyen L.C., Yang D., Nicolaescu V., Best T.J., Ohtsuki T., Chen S.-N., Friesen J.B., Drayman N., Mohamed A., Dann C. (2021). Cannabidiol Inhibits SARS-CoV-2 Replication and Promotes the Host Innate Immune Response. bioRxiv.

[196] Bilal M., Rasheed T., Iqbal H.M., Hu H., Wang W., Zhang X. (2017). Macromolecular agents with antimicrobial potentialities: A drive to combat antimicrobial resistance. Int. J. Biol. Macromol..

[197] Cortes E., Mora J., Márquez E. (2020). Modelling the Anti-Methicillin-Resistant *Staphylococcus Aureus* (MRSA) Activity of Cannabinoids: A QSAR and Docking Study. Crystals.

[198] Hurdle J.G., O’neill A.J., Chopra I., Lee R.E. (2011). Targeting bacterial membrane function: An underexploited mechanism for treating persistent infections. Nat. Rev. Microbiol..

[199] Hyldgaard M., Mygind T., Meyer R.L. (2012). Essential Oils in Food Preservation: Mode of Action, Synergies, and Interactions with Food Matrix Components. Front. Microbiol..

[200] Helander I.M., Alakomi H.-L., Latva-Kala K., Mattila-Sandholm T., Pol I., Smid E.J., Gorris L.G.M., von Wright A. (1998). Characterization of the Action of Selected Essential Oil Components on Gram-Negative Bacteria. J. Agric. Food Chem..

[201] Nazzaro F., Fratianni F., DE Martino L., Coppola R., De Feo V. (2013). Effect of Essential Oils on Pathogenic Bacteria. Pharmaceuticals.

[202] Djilani A., Dicko A., Bouayed J. (2012). The Therapeutic Benefits of Essential Oils. Nutrition, Well-Being and Health.

[203] Jassim S., Naji M. (2003). Novel antiviral agents: A medicinal plant perspective. J. Appl. Microbiol..

[204] Leizer C., Ribnicky D., Poulev A., Dushenkov S., Raskin I. (2000). The Composition of Hemp Seed Oil and Its Potential as an Important Source of Nutrition. J. Nutraceuticals Funct. Med. Foods.

[205] Cowan M.M. (1999). Plant Products as Antimicrobial Agents. Clin. Microbiol. Rev..

[206] Nostro A., Germano M., D’Angelo V., Marino A., Cannatelli M. (2000). Extraction methods and bioautography for evaluation of medicinal plant antimicrobial activity. Lett. Appl. Microbiol..

[207] Valgas C., De Souza S.M., Smânia E.F.A., Smânia A. (2007). Screening methods to determine antibacterial activity of natural products. Braz. J. Microbiol..

[208] Novak J., Franz C. (2003). Composition of the Essential Oils and Extracts of Two Populations ofCannabis sativaL. ssp.spontaneafrom Austria. J. Essent. Oil Res..

[209] Hao X.M., Yang Y., An L.X., Wang J.M., Han L. (2014). Study on Antibacterial Mechanism of Hemp Fiber. Adv. Mater. Res..

[210] Khan B.A., Wang J., Warner P., Wang H. (2014). Antibacterial properties of hemp hurd powder against *E. coli*. J. Appl. Polym. Sci..

[211] Karas J.A., Wong L.J.M., Paulin O.K.A., Mazeh A.C., Hussein M.H., Li J., Velkov T. (2020). The Antimicrobial Activity of Cannabinoids. Antibiotics.

[212] Feldman M., Sionov R., Smoum R., Mechoulam R., Ginsburg I., Steinberg D. (2020). Comparative Evaluation of Combinatory Interaction between Endocannabinoid System Compounds and Poly-L-lysine against Streptococcus mutans Growth and Biofilm Formation. BioMed Res. Int..

[213] ZhengHai Z., Yan D., YanRu J., QingLi Y., ZhenWei L. (2019). Antibacterial activity and stability of extract from hemp (*Cannabis sativa* L.) leaves. J. Food Saf. Qual..

[214] Dhifi W., Bellili S., Jazi S., Bahloul N., Mnif W. (2016). Essential Oils’ Chemical Characterization and Investigation of Some Biological Activities: A Critical Review. Medicines.

[215] Vasudevan K., Stahl V. (2020). Cannabinoids infused mouthwash products are as effective as chlorhexidine on inhibition of total-culturable bacterial content in dental plaque samples. J. Cannabis Res..

[216] Raina S., Thakur A., Sharma A., Pooja D., Minhas A.P. (2019). Bactericidal activity of *Cannabis sativa* phytochemicals from leaf extract and their derived Carbon Dots and Ag@Carbon Dots. Mater. Lett..

[217] Stahl V., Vasudevan K. (2020). Comparison of Efficacy of Cannabinoids versus Commercial Oral Care Products in Reducing Bacterial Content from Dental Plaque: A Preliminary Observation. Cureus.

[218] Guimaraes A.R.D., Peres M.A., Vieira R.D.S., Ferreira R.M., Ramos-Jorge M.L., Apolinario S., Debom A. (2006). Self-perception of side effects by adolescents in a chlorhexidine-fluoride-based preventive oral health program. J. Appl. Oral Sci..

[219] Schirone M., Visciano P., Tofalo R., Suzzi G. (2019). Editorial: Foodborne Pathogens: Hygiene and Safety. Front. Microbiol..

[220] Cabeça T.K., Pizzolitto A.C., Pizzolitto E.L. (2012). Activity of disinfectants against foodborne pathogens in suspension and adhered to stainless steel surfaces. Braz. J. Microbiol..

[221] Langsrud S., Sidhu M.S., Heir E., Holck A.L. (2003). Bacterial disinfectant resistance—a challenge for the food industry. Int. Biodeterior. Biodegrad..

[222] Park K.M., Yoon S.-G., Choi T.-H., Kim H.J., Park K.J., Koo M. (2020). The Bactericidal Effect of a Combination of Food-Grade Compounds and their Application as Alternative Antibacterial Agents for Food Contact Surfaces. Foods.

[223] Colagiorgi A., Bruini I., Di Ciccio P.A., Zanardi E., Ghidini S., Ianieri A. (2017). Listeria monocytogenes Biofilms in the Wonderland of Food Industry. Pathogens.

[224] Krawczyk-Balska A., Markiewicz Z. (2015). The intrinsic cephalosporin resistome of Listeria monocytogenes in the context of stress response, gene regulation, pathogenesis and therapeutics. J. Appl. Microbiol..

[225] Doulgeraki A.I., Di Ciccio P., Ianieri A., Nychas G.-J. (2017). Methicillin-resistant food-related Staphylococcus aureus: A review of current knowledge and biofilm formation for future studies and applications. Res. Microbiol..

[226] Kurtzman C.P. (2011). Torulaspora Lindner (1904). The Yeasts.

[227] Kuanyshev N., Adamo G.M., Porro D., Branduardi P. (2017). The spoilage yeastZygosaccharomyces bailii: Foe or friend?. Yeast.

[228] Pasquali F., Schinzari M., Lucchi A., Mandrioli M., Toschi T.G., De Cesare A., Manfreda G. (2020). Preliminary data on the antimicrobial effect of *Cannabis sativa* L. variety Futura 75 against food-borne pathogens in vitro as well as against naturally occurring microbial populations on minced meat during storage. Ital. J. Food Saf..

[229] Doehlemann G., Ökmen B., Zhu W., Sharon A. (2017). Plant Pathogenic Fungi. Fungal Kingd..

[230] Ingold C.T., Hudson H.J., Ingold C.T. (1993). Fungi as Plant Pathogens. The Biology of Fungi.

[231] Yang J., Hsiang T., Bhadauria V., Chen X.-L., Li G. (2017). Plant Fungal Pathogenesis. BioMed Res. Int..

[232] Wightwick A., Walters R., Allinson G., Reichman S., Menzies S.R.A.N., Carisse O. (2010). Environmental Risks of Fungicides Used in Horticultural Production Systems. Fungicides.

[233] Zubrod J.P., Bundschuh M., Arts G., Brühl C.A., Imfeld G., Knäbel A., Payraudeau S., Rasmussen J.J., Rohr J., Scharmüller A. (2019). Fungicides: An Overlooked Pesticide Class?. Environ. Sci. Technol..

[234] Tapwal A., Nisha, Garg S., Gautam N., Kumar R. (2011). In Vitro antifungal potency of plant extracts against five phytopathogens. Braz. Arch. Biol. Technol..

[235] Garcia-Aroca T., Doyle V., Singh R., Price T., Collins K. (2018). First Report of Curvularia Leaf Spot of Corn, Caused by Curvularia lunata, in the United States. Plant Health Prog..

[236] Haroun N.E., Elamin S.E., Mahgoub B.M., Elssidig M.A., Mohammed E.H. (2015). Leaf blight: A new disease of Xanthium strumarium L. caused by Curvularia lunata and Drechslera spicifera in Sudan. Int. J. Curr. Microbiol. Appl. Sci..

[237] Msikita W., Baimey H., James B.D. (2007). Severity of Curvularia Stem Blight Disease of Cassava in West Africa. Plant Dis..

[238] Verma V.S., Gupta V.K. (2010). First Report of Curvularia lunata Causing Root Rot of Strawberry in India. Plant Dis..

[239] Farooq M.A., Iqbal U., Iqbal S.M., Afzal R., Rasool A. (2010). Invitro evaluation of different plant extracts on mycelial growth of sclerotium rolfsii the cause of root rot of sugar beet. Mycopath.

[240] Pal G.K., Kumar B., Shani S.K. (2013). Antifungal activity of some seed extracts against seed-borne phytopathogenic fungi *Alternaria* Spp.. Int. J. Univers. Pharm. Life Sci..

[241] Aphajal M., Jaish B.M. (2018). In vitro Antifungal activity of somee higher plant extracts against Alternaria brassicae (Berk.) sacc. and A. brassicicola (Schw.) Wiltsh. Bull. Pure Appl. Sci. Bot..

[242] Thomma B.P.H.J. (2003). Alternariaspp.: From general saprophyte to specific parasite. Mol. Plant Pathol..

[243] Charkowski A.O. (2015). Biology and control of Pectobacterium in potato. Am. J. Potato Res..

[244] Bao Q., Liu H., Fu K., Zhang C., Wang C., Feng Y., Yang L., Lao H., Ren Q. (2016). Hemp Bast Fiber Extract with Antibacterial Activity, Preparation Method and Application of Hemp Bast Fiber Extract 2014.

[245] Cassano R., Trombino S., Ferrarelli T., Nicoletta F.P., Mauro M.V., Giraldi C., Picci N. (2013). Hemp fiber (*Cannabis sativa* L.) derivatives with antibacterial and chelating properties. Cellulose.

[246] Fang G.L. (2006). Surgical Sewing Free Zipper Made of Antibiotic Material Hemp Fiber 2005.

[247] Andre C.M., Hausman J.-F., Guerriero G. (2016). Cannabis sativa: The Plant of the Thousand and One Molecules. Front. Plant Sci..

[248] Khan B.A., Warner P., Wang H. (2014). Antibacterial Properties of Hemp and Other Natural Fibre Plants: A Review. BioResources.

[249] Stasiłowicz A., Tomala A., Podolak I., Cielecka-Piontek J. (2021). *Cannabis sativa* L. as a Natural Drug Meeting the Criteria of a Multitarget Approach to Treatment. Int. J. Mol. Sci..

[250] Stott C.G., Guy G.W. (2004). Cannabinoids for the pharmaceutical industry. Euphytica.

[251] McKenna G.J. (2014). The Current Status of Medical Marijuana in the United States. Hawaii J. Med. Public Health.

[252] Koltai H., Poulin P., Namdar D. (2019). Promoting cannabis products to pharmaceutical drugs. Eur. J. Pharm. Sci..

[253] Benito S.B., Seijo-Vila M., Caro-Villalobos M., Tundidor I., Andradas C., García-Taboada E., Wade J., Smith S., Guzmán M., Pérez-Gómez E. (2018). Appraising the “entourage effect”: Antitumor action of a pure cannabinoid versus a botanical drug preparation in preclinical models of breast cancer. Biochem. Pharmacol..

[254] Nahtigal I., Blake A., Hand A., Florentinus-Mefailoski A., Sohi H.H., Friedberg J., Merrick J. (2016). The pharmacological properties of cannabis. Pain Management Yearbook 2016.

[255] Sharma P., Murthy P., Bharath M.S. (2012). Chemistry, Metabolism, and Toxicology of Cannabis: Clinical Implications. Iran. J. Psychiatry.

[256] Tamilselvan N., Thirumalai T., Shyamala P., David E. (2014). A review on some poisonous plants and their medicinal values. J. Acute Dis..

[257] Borgelt L.M., Franson K.L., Nussbaum A., Wang G.S. (2013). The Pharmacologic and Clinical Effects of Medical Cannabis. Pharmacother. J. Hum. Pharmacol. Drug Ther..

[258] Pertwee R.G., Howlett A.C., Abood M.E., Alexander S.P., Di Marzo V., Elphick M.R., Greasley P.J., Hansen H.S., Kunos G., Mackie K. (2010). International Union of Basic and Clinical Pharmacology. LXXIX. Cannabinoid Receptors. Pharmacol. Rev..

[259] Uliss D.B., Dalzell H.C., Handrick G.R., Howes J.F., Razdan R.K. (1975). Hashish. Importance of the phenolic hydroxyl group in tetrahydrocannabinols. J. Med. Chem..

[260] Vaughn D., Kulpa J., Paulionis L. (2020). Preliminary Investigation of the Safety of Escalating Cannabinoid Doses in Healthy Dogs. Front. Vet. Sci..

[261] Deiana S., Watanabe A., Yamasaki Y., Amada N., Arthur M., Fleming S., Woodcock H., Dorward P., Pigliacampo B., Close S. (2011). Plasma and brain pharmacokinetic profile of cannabidiol (CBD), cannabidivarine (CBDV), Δ9-tetrahydrocannabivarin (THCV) and cannabigerol (CBG) in rats and mice following oral and intraperitoneal administration and CBD action on obsessive–compulsive behaviour. Psychopharmacology.

[262] Satyal P., Setzer W.N. (2014). Chemotyping and Determination of Antimicrobial, Insecticidal, and Cytotoxic Properties of Wild- Grown Cannabis sativa from Nepal. J. Med. Act. Plants J. Med. Act. Plants J. Med. Act. Plants.

[263] Zuardi A.W., Crippa J.A.S., Hallak J.E., Bhattacharyya S., Atakan Z., Martin-Santos R., McGuire P., Guimaraes F.S. (2012). A Critical Review of the Antipsychotic Effects of Cannabidiol: 30 Years of a Translational Investigation. Curr. Pharm. Des..

[264] Thompson G.R., Rosenkrantz H., Schaeppi U.H., Braude M.C. (1973). Comparison of acute oral toxicity of cannabinoids in rats, dogs and monkeys. Toxicol. Appl. Pharmacol..

[265] Beaulieu P. (2005). Toxic effects of cannabis and cannabinoids: Animal data. Pain Res. Manag..

[266] Di L., Kerns E., Carter G. (2009). Drug-Like Property Concepts in Pharmaceutical Design. Curr. Pharm. Des..

[267] Kerns E.H., Di L., Kerns E.H., Di L. (2016). Advantages of Good Drug-like Properties. Drug-Like Properties: Concepts, Structure Design and Methods from ADME to Toxicity Optimization.

[268] Landmark C.J., Brandl U. (2020). Pharmacology and drug interactions of cannabinoids. Epileptic Disord..

[269] Prandi C., Blangetti M., Namdar D., Koltai H. (2018). Structure-Activity Relationship of Cannabis Derived Compounds for the Treatment of Neuronal Activity-Related Diseases. Molecules.

[270] Furqan T., Batool S., Habib R., Shah M., Kalasz H., Darvas F., Kuca K., Nepovimova E., Batool S., Nurulain S.M. (2020). Cannabis Constituents and Acetylcholinesterase Interaction: Molecular Docking, In Vitro Studies and Association with CNR1 rs806368 and ACHE rs17228602. Biomolecules.

[271] Filloux F.M. (2015). Cannabinoids for pediatric epilepsy? Up in smoke or real science?. Transl. Pediatr..

[272] Desa S., Osman A., Hyslop R. (2017). In Silico Assessment of Drug-Like Properties of Phytocannabinoids in Cannabis Sativa. Educ. J. Sci. Math. Technol..

[273] Wiley J.L., Martin B.R. (2003). Cannabinoid pharmacological properties common to other centrally acting drugs. Eur. J. Pharmacol..

[274] New Antimicrobial Data and Conference Presentation. https://www.asx.com.au/asxpdf/20200908/pdf/44mf662gw8dbkd.pdf.

[275] Kocis P., Vrana K.E. (2020). Delta-9-Tetrahydrocannabinol and Cannabidiol Drug-Drug Interactions. Med Cannabis Cannabinoids.

[276] Parmar J.R., Forrest B.D., Freeman R.A. (2015). Medical marijuana patient counseling points for health care professionals based on trends in the medical uses, efficacy, and adverse effects of cannabis-based pharmaceutical drugs. Res. Soc. Adm. Pharm..

[277] Cabral G.A., Pettit D.A.D. (1998). Drugs and immunity: Cannabinoids and their role in decreased resistance to infectious disease. J. Neuroimmunol..

[278] FDA Grants BTX 1801 Qualified Infectious Disease Product Designation Status. https://yourir.info/resources/3f148bb5dfccdf8f/announcements/bot.asx/6A976285/BOT_FDA_Grants_BTX_1801_QIDP_Designation_Status.pdf?embed=1.

